# The association between gene polymorphisms in voltage-gated potassium channels Kv2.1 and Kv4.2 and susceptibility to autism spectrum disorder

**DOI:** 10.3389/fpsyt.2022.994166

**Published:** 2023-01-23

**Authors:** Zehui Liu, Xiaolei Yang, Peiwen Guo, Feng Wang, Wei Xia, Yuxin Chen, Mingyang Zou, Caihong Sun

**Affiliations:** ^1^Department of Children’s and Adolescent Health, Public Health College, Harbin Medical University, Harbin, China; ^2^Department of Preventive Medicine, School of Public Health, Qiqihar Medical University, Qiqihar, China; ^3^Faculty of Arts and Science, University of Toronto, Toronto, ON, Canada

**Keywords:** autism spectrum disorder, Kv2.1, Kv4.2, single nucleotide polymorphisms, susceptibility

## Abstract

**Background:**

Autism spectrum disorder (ASD) is a heritable form of neurodevelopmental disorder that arises through synaptic dysfunction. Given the involvement of voltage-gated potassium (Kv) channels in the regulation of synaptic plasticity, we aimed to explore the relationship between the genetic variants in the *KCNB1* and *KCND2* genes (encoding Kv2.1 and Kv4.2, respectively) and the risk of developing ASD.

**Methods:**

A total of 243 patients with ASD and 243 healthy controls were included in the present study. Sixty single nucleotide polymorphisms (SNPs) (35 in *KCNB1* and 25 in *KCND2*) were genotyped using the Sequenom Mass Array.

**Results:**

There were no significant differences in the distribution of allele frequencies and genotype frequencies in *KCNB1* between cases and controls. However, the differences were significant in the allelic distribution of *KCND2 rs1990429* (*p*_*Bonferroni*_ < 0.005) and *rs7793864* (*p*_*Bonferroni*_ < 0.005) between the two groups. *KCND2 rs7800545* (*p*_*FDR*_ = 0.045) in the dominant model and *rs1990429* (*p*_*FDR*_ < 0.001) and *rs7793864* (*p*_*FDR*_ < 0.001) in the over-dominant model were associated with ASD risk. The G/A genotype of *rs1990429* in the over-dominant model and the G/A–G/G genotype of *rs7800545* in the dominant model were correlated with lower severity in the Autism Diagnostic Interview-Revised (ADI–R) restricted repetitive behavior (RRB) domain.

**Conclusion:**

Our results provide evidence that *KCND2* gene polymorphism is strongly associated with ASD susceptibility and the severity of RRB.

## 1. Introduction

Autism spectrum disorder (ASD) is a neurodevelopmental disease characterized by impaired social interaction and repetitive/stereotyped behaviors. It is reported that the overall prevalence of ASD is 23.0 per 1,000 children aged 8 years in the United States, and the estimated prevalence is 7.0 per 1,000 children aged 6–12 years in China (1, 2). This alarming situation implies that ASD is being increasingly recognized as a major health burden. Generally, it is assumed that ASD involves strong genetic components, whose heritability is estimated to be 83% (3). Our previous study found that 2,174 candidate genes were closely related to ASD, based on whole-genome sequencing. These genes included 14,310 single nucleotide polymorphisms (SNPs), the majority of which play important roles in the regulation of synaptic development and plasticity (4). Numerous studies have confirmed that alterations in synaptic plasticity and neuronal excitation/inhibition imbalance contribute to the major etiology of ASD.

Potassium channels, broadly distributed on neuronal cell membranes, are crucial determinants of synaptic plasticity and neuronal excitability. Accumulating evidence shows that genetic mutations in potassium channels inducing alterations of K^+^ current change the synaptic plasticity and information processing capacity of the brain, thus potentially impairing brain connectivity and function in the early life of individuals with ASD (5). Voltage-gated potassium (Kv) channels are important modulators involved in regulating neuronal excitability, mainly including transient outward potassium channels and delayed rectifier potassium channels. Recently, the strong genetic associations between Kv channels and ASD have been partially explained. The expression level of Kv10.2 was significantly reduced in the hippocampus of VPA-induced rats (a model of ASD), and autism-like behaviors, such as stereotypical behaviors and impaired social and exploratory abilities, were effectively ameliorated after upregulation of Kv10.2 expression by lentivirus injection in the hippocampus (6). *KCNC1* (encoding Kv3.1) knockout mice show deficits in social interaction and hyperactivity (7). The Kv7 mutant has been proposed to be potentially pathogenic in autism, owing to altered action potential generation (8). The above-mentioned evidence shows that deleterious mutations in Kv channels impact ASD pathogenesis directly or indirectly, which may cause impairments in social interaction and cognitive function.

Kv2.1 (encoded by *KCNB1*) is the principal delayed rectifier potassium channel, which alters the action potential threshold and firing frequency (9). Kv4.2 (encoded by *KCND2*) is a major predominant transient outward potassium channel, which regulates neuronal signaling by regulating back-propagating action potentials, synaptic integration, and long-term potentiation (10). Kv2.1 and Kv4.2 are expressed at high levels throughout the various hippocampal subfields, especially in the CA1 dendritic field (9, 10). The hippocampus is an important brain region in ASD, which implies that these two Kv channels might be central nodes of dysfunction in ASD. Research has found that 7 of 19 mutants in *KCNB1*, including S202F, R306C, R312H, W370R, V378A, P385T, and F416L, altered the activity of the voltage-dependence channel, current density, and conductance, which caused epileptic spasms and autism-like developmental phenotypes (11). Zhang et al. (12) showed that strong gating impairment, associated with substitutions of V404L, or V404M in *KCND2*, increased susceptibility to autism and epileptic seizures. Numerous studies have shown that ASD and epilepsy often co-exist as parallel syndromes. Even in the absence of epileptic seizures, roughly 4–86% of individuals with autism presented abnormal electroencephalogram patterns (13). The frequent comorbidity between ASD and epilepsy implies shared underlying neurological abnormalities. It is widely recognized that *KCNB1* and *KCND2* are the main causes of developmental and epileptic encephalopathy and neurodevelopmental disability (12, 14). Therefore, we speculated that *KCNB1* and *KCND2* may be risk genes for autism.

To investigate whether *KCNB1* and *KCND2* gene polymorphisms are related to ASD risk in the Chinese Han population, we identified the SNPs in the *KCNB1* and *KCND2* genes between patients with autism and control individuals by allele frequencies, genotype frequencies, and haplotype analyses. Furthermore, we evaluated the relationship between the SNPs and the severity of ASD symptoms. Our results offer supporting evidence for the involvement of Kv channels in the etiology of ASD.

## 2. Materials and methods

### 2.1. Participants

The case-control study included 243 pairs of subjects: the ASD patients were recruited from the Child Development and Behavior Research Center of Harbin Medical University, Harbin, China, and the controls were selected from kindergartens, junior schools in Harbin, China. All the subjects were of Chinese Han ethnicity, aged between 2 and 10 years. The diagnosis was confirmed by two professional clinicians according to DSM–V. Most participants with autism were evaluated using Autism Diagnostic Observation Schedule (ADOS) or Autism Diagnostic Interview-Revised (ADI–R), which are currently the gold standard for assessing ASD severity. Participants were excluded if they had other neuropsychiatric disorders or known genetic disorders, such as epilepsy, tuberous sclerosis, intellectual disability, or fragile X syndrome.

The study received ethics approval from the Institutional Review Board of Harbin Medical University for Medical Sciences (Ethics approval number: HMUIRB2012007). The research was performed in accordance with the Declaration of Helsinki principles. All participants or their relatives provided written informed consent.

### 2.2. Clinical evaluation

The ADOS and ADI–R were assessed to obtain information about autism-specific behaviors and symptoms. Each patient was scored by trained examiners through structured clinical interview with their patients or guardians. Ultimately, of the 243 cases, 166 (68.3%) individuals completed the ADOS assessment and 162 (66.7%) individuals completed the ADI–R assessment. Sample characteristics are provided in Supplementary Table 1. ADOS is a semi-structured observational assessment, which is organized into four modules to assess participants’ social and communication abilities in a standardized context. The calibrated severity score (CSS) is a standardized score of the relative severity of autism-specific behaviors, less influenced by demographic and developmental factors (15). This diagnostic algorithm consists of social affect (SA) and restricted repetitive behavior (RRB) domains, which are consistent with DSM–V (16). A detailed description of procedures for deriving the ADOS–CSS, SA–CSS, and RRB–CSS can be found in the original study (15, 17). ADI–R is an anamnestic interview with ASD parents or caregivers, mainly providing information on early development. The interview encompasses three behavioral domains: social, communication, and RRB. Higher scores indicate greater impairment. The intelligence quotient (IQ) scores were derived from the Wechsler Intelligence Scale for Children or the Peabody Picture Vocabulary Test.

### 2.3. SNP selection and genotyping

Tag SNPs in *KCNB1* and *KCND2* genes were selected from the Chinese Han in the Beijing panel of HapMap Project Phase II, including 2,000–bp upstream regions at least. Tag SNP selection was performed using the Tagger program incorporated in Haploview v.4.2 software (Broad Institute of Massachusetts Institute of Technology and Harvard, Cambridge, MA, USA) according to the following criteria: minor allele frequency ≥ 0.05 and pairwise tagging (*r*^2^ ≥ 0.8). Finally, 57 tag SNPs (32 in *KCNB1* and 25 in *KCND2* that captured from more than 98 and 216 initially identified SNPs, respectively) were selected for genotyping along with three additional single nucleotide variants in *KCNB1* (*rs587777850*, *rs587777849*, and *rs587777848*) that have been linked to autism phenotypes in the previous study (18). Mutation information and location for tag SNPs are shown in Supplementary Tables 2, 3.

All the blood samples were collected in venous blood collection tubes containing EDTA. Genomic DNA was extracted from blood cells by the Qiagen QIAamp DNA Mini kit (Qiagen, Hilden, Germany) following the manufacturer’s instructions. Quality assessment and concentration estimation of DNA were done using gel electrophoresis and NanoDrop 2000 spectrophotometer (Thermo Fisher Scientific, Waltham, MA, United States). SNP genotyping was done using the MassArray platform (Sequenom, San Diego, CA, United States) with primers listed in Supplementary Tables 4, 5. Genotyping quality control for all 60 SNPs was tested using blinded duplicate genotyping for 30 random samples, with reproducibility of 100%.

### 2.4. Statistical analyses

Hardy-Weinberg equilibrium (HWE) among controls, Linkage disequilibrium (LD) and haplotype construction were determined with Haploview v.4.2 software. Allelic associations were performed with logistic regression using SPSS v.21.0 software (SPSS Inc., Chicago, IL, United States). The odds ratio (OR) and 95% confidence interval (CI) were calculated in different genetic models using SNPstats software^1^. Genotype associations were estimated according to genetic models of codominant, dominant, recessive, over-dominant, and log-additive, with further adjustment for gender and age. The Akaike information criterion (AIC) and Bayesian information criterion (BIC) were used for model selection, with the lower AIC and BIC indicating the better models. To avoid false-positive results in multiple comparisons, *p*-values were obtained by either FDR corrected using the Benjamini-Hochberg procedure or Bonferroni correction. Potential associations between genotype and symptom phenotype were evaluated by one-way analysis of variance with adjustment for age, sex, and IQ. All *p*-values were two tailed, with the significance level set at α = 0.05.

## 3. Results

### 3.1. General demographics

There were 243 individuals (39 girls and 204 boys) in the ASD group and 243 (51 girls and 192 boys) in the control group; the mean ages were 5.19 ± 1.96 and 4.96 ± 0.97 years, respectively. The groups did not differ statistically in age (*t* = 1.643, *p* = 0.101) and gender (χ^2^ = 1.964, *p* = 0.161).

### 3.2. Links between SNPs and ASD risk

There were a total of 7 SNPs out of the 35 SNPs that were removed in *KCNB1*: the *rs237454*, *rs490840*, and *rs802952* call rates were lower than 90%; *rs587777850*, *rs58777749*, and *rs58777748* showed no dimorphism among participants; *rs1961192* deviated from HWE in the control group (*p* < 0.01; Table 1). In total, 4 out of 25 SNPs were excluded in *KCND2*: the *rs802359* and *rs12673992* call rates were lower than 90%; *rs2191736* and *rs2189977* deviated from HWE in the control group (*p* < 0.01; Table 2).

**TABLE 1:** Characteristics of *KCNB1* single nucleotide polymorphisms (SNPs).

	SNPs ID	Gene	Genomic position (bp)	Genic position	Reference allele^a^	Call rate%	MAF^b^	HWE^b^ (*P*)
1	*rs1051295*	*KCNB1*	49372368	intron variant, utr variant 3 prime	A	95.3	0.442	0.362
2	*rs6019774*	*KCNB1*	49375641	intron	T	99.4	0.060	0.405
3	*rs2426154*	*KCNB1*	49385458	intron	T	97.9	0.419	0.089
4	*rs4810952*	*KCNB1*	49389638	intron	T	98.8	0.442	0.233
5	*rs1961192*	*KCNB1*	49389770	intron	G	98.8	0.192	<0.001*
6	*rs9636516*	*KCNB1*	49390663	intron	A	94.0	0.455	0.354
7	*rs756529*	*KCNB1*	49394471	intron	A	99.0	0.415	1.000
8	*rs7348799*	*KCNB1*	49405190	intron	T	99.8	0.062	0.449
9	*rs6067087*	*KCNB1*	49410131	intron	A	97.9	0.491	0.455
10	*rs6019820*	*KCNB1*	49410790	intron	G	98.6	0.399	0.937
11	*rs237454*	*KCNB1*	49413854	intron	A	**67.3**	0.379	0.086
12	*rs237459*	*KCNB1*	49415093	intron	T	98.2	0.440	0.394
13	*rs237477*	*KCNB1*	49440911	intron	T	99.0	0.324	0.856
14	*rs3787318*	*KCNB1*	49441530	intron	T	99.2	0.071	0.661
15	*rs742759*	*KCNB1*	49444608	intron	G	99.6	0.138	0.994
16	*rs237478*	*KCNB1*	49449322	intron	C	99.4	0.459	0.198
17	*rs13044742*	*KCNB1*	49457703	intron	A	96.3	0.120	0.883
18	*rs572845*	*KCNB1*	49460077	intron	G	99.8	0.128	0.142
19	*rs610412*	*KCNB1*	49461665	intron	A	99.4	0.162	0.749
20	*rs6019855*	*KCNB1*	49465446	intron	T	98.8	0.269	0.390
21	*rs10485612*	*KCNB1*	49468115	intron	A	98.4	0.072	0.672
22	*rs802950*	*KCNB1*	49469295	intron	C	99.0	0.176	1.000
23	*rs490840*	*KCNB1*	49469418	intron	T	**31.7**	0.319	0.437
24	*rs802952*	*KCNB1*	49469732	intron	T	32.9	0.190	1.000
25	*rs653070*	*KCNB1*	49470871	intron	C	98.6	0.335	0.551
26	*rs4809745*	*KCNB1*	49471334	intron	G	98.4	0.104	0.484
27	*rs552068*	*KCNB1*	49471461	intron	A	99.2	0.438	0.908
28	*rs6125656*	*KCNB1*	49474242	intron	G	99.0	0.067	0.658
29	*rs477135*	*KCNB1*	49475917	intron	T	98.2	0.179	0.012
30	*rs566604*	*KCNB1*	49478053	intron	A	99.4	0.164	0.588
31	*rs7269864*	*KCNB1*	49480071	intron	T	99.2	0.110	0.283
32	*rs553213*	*KCNB1*	49481027	intron	A	97.9	0.357	0.480
33	*rs587777850*	*KCNB1*	49374425	intron variant, missense	C	99.0	0.002	1.000
34	*rs587777849*	*KCNB1*	49374439	intron variant, missense	G	98.8	0.000	1.000
35	*rs587777848*	*KCNB1*	49374519	intron variant, missense	G	98.8	0.000	1.000

MAF, minor allele frequency; HWE, hardy–weinberg equilibrium. *p < 0.01, ^a^determined by most frequent allele among controls; ^b^among controls (*n* = 243). The bold represent the statistically significant correlations (*p* < 0.05), or meaningful models or values.

**TABLE 2:** Characteristics of *KCND2* single nucleotide polymorphisms (SNPs).

	SNPs ID	Gene	Genomic position (bp)	Genic position	Reference allele^a^	Call rate	MAF^b^	HWE^b^ (*P*)
1	*rs1990429*	*KCND2*	120293793	intron variant, utr variant 3 prime	G	99.0	0.123	0.033
2	*rs7800545*	*KCND2*	120303057	intron	A	99.0	0.104	0.970
3	*rs2191736*	*KCND2*	120343577	intron	A	94.9	0.200	<0.001*
4	*rs17142666*	*KCND2*	120350103	intron	G	97.5	0.331	0.991
5	*rs7793864*	*KCND2*	120367201	intron	T	98.2	0.056	0.922
6	*rs7810357*	*KCND2*	120385502	intron	G	98.4	0.071	1.000
7	*rs7793037*	*KCND2*	120431403	intron	A	98.4	0.485	0.732
8	*rs2192373*	*KCND2*	120452784	intron	C	99.0	0.063	0.758
9	*rs802359*	*KCND2*	120509095	intron	A	**63.0**	0.470	0.010
10	*rs802372*	*KCND2*	120529262	intron	A	98.6	0.471	0.200
11	*rs1527650*	*KCND2*	120603522	intron	T	98.6	0.038	0.568
12	*rs10278347*	*KCND2*	120619780	intron	C	98.2	0.471	0.307
13	*rs2402539*	*KCND2*	120628249	intron	T	97.3	0.489	0.317
14	*rs6979618*	*KCND2*	120629117	intron	A	98.6	0.483	0.323
15	*rs7779895*	*KCND2*	120629812	intron	C	92.8	0.417	0.578
16	*rs17142875*	*KCND2*	120632817	intron	A	99.2	0.477	0.070
17	*rs4727911*	*KCND2*	120632910	intron	G	98.2	0.498	0.160
18	*rs7795646*	*KCND2*	120683673	intron	A	98.4	0.454	0.227
19	*rs2896298*	*KCND2*	120687230	intron	T	98.8	0.411	0.034
20	*rs1072198*	*KCND2*	120687295	intron	T	98.8	0.069	0.611
21	*rs17142891*	*KCND2*	120698554	intron	G	97.7	0.418	0.061
22	*rs2189977*	*KCND2*	120704164	intron	C	99.4	0.367	<0.001*
23	*rs11983106*	*KCND2*	120719673	intron	G	99.0	0.040	1.000
24	*rs12673992*	*KCND2*	120732769	intron	A	**39.5**	0.474	1.000
25	*rs727228*	*KCND2*	120749594	utr variant 3 prime	T	99.2	0.416	0.394

MAF, minor allele frequency; HWE, hardy–weinberg equilibrium. *p < 0.01; ^a^determined by most frequent allele among controls; ^b^among controls (*n* = 243). The bold represent the statistically significant correlations (*p* < 0.05), or meaningful models or values.

Tables 3, 4 show the allele frequency distributions in cases and controls. Allele frequencies for *KCNB1* were not significantly different between the two groups. Five tag SNPs showed statistically significant differences in *KCND2* after adjusting for age and sex (*p* < 0.05). The *rs1990429* A allele (OR = 0.53, 95% CI = 0.34–0.82, *p* = 0.004), *rs7800545* G allele (OR = 0.53, 95% CI = 0.33–0.85, *p* = 0.009), *rs7793864* A allele (OR = 0.23, 95% CI = 0.10–0.53, *p* = 0.001), *rs7810357* A allele (OR = 0.50, 95% CI = 0.28–0.89, *p* = 0.019), and *rs6979618* G allele (OR = 0.74, 95% CI = 0.58–0.96, *p* = 0.024) were associated with a lower risk of ASD. Of particular concern, *rs1990429* and *rs7793864* remained positive after Bonferroni correction (*p*_*Bonferroni*_ < 0.005).

**TABLE 3:** Distribution of allele frequencies of *KCNB1* single nucleotide polymorphisms (SNPs) in cases and controls (n, %).

	SNPs ID	Gene	Allele	Control (243)	Case (243)	*P* ^#^	Adjusted OR (95% CI)^#^
1	*rs1051295*	*KCNB1*	A	258 (55.8%)	246 (53.0%)	0.369	1.00
			G	204 (44.2%)	218 (47.0%)		1.13 (0.87–1.46)
2	*rs6019774*	*KCNB1*	T	453 (94.0%)	442 (91.3%)	0.135	1.00
			C	29 (6.0%)	42 (8.7%)		1.46 (0.89–2.38)
3	*rs2426154*	*KCNB1*	T	273 (58.1%)	283 (58.7%)	0.746	1.00
			C	197 (41.9%)	199 (41.3%)		0.96 (0.74–1.24)
4	*rs4810952*	*KCNB1*	T	269 (55.8%)	286 (59.8%)	0.177	1.00
			C	213 (44.2%)	192 (40.2%)		0.84 (0.65–1.08)
5	*rs9636516*	*KCNB1*	A	243 (54.5%)	249 (53.2%)	0.789	1.00
			G	203 (45.5%)	219 (46.8%)		1.04 (0.80–1.35)
6	*rs756529*	*KCNB1*	A	281 (58.5%)	298 (61.8%)	0.253	1.00
			G	199 (41.5%)	184 (38.2%)		0.86 (0.66–1.11)
7	*rs7348799*	*KCNB1*	T	454 (93.8%)	445 (91.6%)	0.200	1.00
			C	30 (6.2%)	41 (8.4%)		1.38 (0.84–2.25)
8	*rs6067087*	*KCNB1*	A	239 (50.9%)	244 (50.6%)	0.999	1.00
			G	231 (49.1%)	238 (49.4%)		1.00 (0.78–1.29)
9	*rs6019820*	*KCNB1*	G	286 (60.1%)	304 (63.1%)	0.338	1.00
			A	190 (39.9%)	178 (36.9%)		0.88 (0.68–1.14)
10	*rs237459*	*KCNB1*	T	269 (56.0%)	268 (56.5%)	0.885	1.00
			C	211 (44.0%)	206 (43.5%)		0.98 (0.76–1.27)
11	*rs237477*	*KCNB1*	T	326 (67.6%)	327 (68.1%)	0.982	1.00
			C	156 (32.4%)	153 (31.9%)		1.00 (0.76–1.31)
12	*rs3787318*	*KCNB1*	T	446 (92.9%)	435 (89.9%)	0.116	1.00
			C	34 (7.1%)	49 (10.1%)		1.45 (0.91–2.29)
13	*rs742759*	*KCNB1*	G	417 (86.2%)	404 (83.5%)	0.294	1.00
			A	67 (13.8%)	80 (16.5%)		1.21 (0.85–1.72)
14	*rs237478*	*KCNB1*	C	261 (54.1%)	262 (54.1%)	0.911	1.00
			T	221 (45.9%)	222 (45.9%)		1.02 (0.79–1.31)
15	*rs13044742*	*KCNB1*	A	419 (88.0%)	408 (88.7%)	0.695	1.00
			T	57 (12.0%)	52 (11.3%)		0.92 (0.62–1.38)
16	*rs572845*	*KCNB1*	G	424 (87.2%)	427 (88.2%)	0.571	1.00
			A	62 (12.8%)	57 (11.8%)		0.89 (0.61–1.32)
17	*rs610412*	*KCNB1*	A	404 (83.8%)	401 (82.9%)	0.795	1.00
			C	78 (16.2%)	83 (17.1%)		1.05 (0.74–1.47)
18	*rs6019855*	*KCNB1*	T	348 (73.1%)	344 (71.1%)	0.546	1.00
			G	128 (26.9%)	140 (28.9%)		1.09 (0.82–1.45)
19	*rs10485612*	*KCNB1*	A	440 (92.8%)	434 (90.0%)	0.140	1.00
			G	34 (7.2%)	48 (10.0%)		1.41 (0.89–2.24)
20	*rs802950*	*KCNB1*	C	394 (82.4%)	408 (84.3%)	0.412	1.00
			A	84 (17.6%)	76 (15.7%)		0.87 (0.62–1.22)
21	*rs653070*	*KCNB1*	C	315 (66.5%)	319 (65.9%)	0.976	1.00
			T	159 (33.5%)	165 (34.1%)		1.00 (0.77–1.32)
22	*rs4809745*	*KCNB1*	G	430 (89.6%)	413 (86.8%)	0.216	1.00
			A	50 (10.4%)	63 (13.2%)		1.28 (0.86–1.91)
23	*rs552068*	*KCNB1*	A	271 (56.2%)	259 (53.7%)	0.564	1.00
			C	211 (43.8%)	223 (46.3%)		1.08 (0.84–1.39)
24	*rs6125656*	*KCNB1*	G	446 (93.3%)	443 (91.5%)	0.311	1.00
			A	32 (6.7%)	41 (8.5%)		1.28 (0.79–2.08)
25	*rs477135*	*KCNB1*	T	391 (82.1%)	377 (78.9%)	0.250	1.00
			A	85 (17.9%)	101 (21.1%)		1.21 (0.88–1.67)
26	*rs566604*	*KCNB1*	A	403 (83.6%)	384 (79.3%)	0.111	1.00
			G	79 (16.4%)	100 (20.7%)		1.31 (0.94–1.81)
27	*rs7269864*	*KCNB1*	T	427 (89.0%)	421 (87.0%)	0.404	1.00
			C	53 (11.0%)	63 (13.0%)		1.18 (0.80–1.75)
28	*rs553213*	*KCNB1*	A	305 (64.3%)	292 (61.1%)	0.383	1.00
			G	169 (35.7%)	186 (38.9%)		1.13 (0.86–1.47)

^#^*P* value and OR adjusted by age and sex.

**TABLE 4:** Distribution of allele frequencies of *KCND2* single nucleotide polymorphisms (SNPs) in cases and controls (n, %).

	SNPs ID	Gene	Allele	Control (243)	Case (243)	*P* ^#^	Adjusted OR (95% CI)^#^
1	*rs1990429*	*KCND2*	G	419 (87.7%)	450 (93.0%)	0.004*	1.00
			A	59 (12.3%)	34 (7.0%)		**0.53 (0.34**–**0.82)**
2	*rs7800545*	*KCND2*	A	432 (89.6%)	452 (94.2%)	**0.009**	1.00
			G	50 (10.4%)	28 (5.8%)		**0.53 (0.33**–**0.85)**
3	*rs17142666*	*KCND2*	G	314 (66.8%)	320 (66.9%)	0.920	1.00
			A	156 (33.2%)	158 (33.1%)		0.99 (0.75–1.29)
4	*rs7793864*	*KCND2*	T	454 (94.2%)	475 (98.5%)	0.001*	1.00
			A	28 (5.8%)	7 (1.5%)		**0.23 (0.10**–**0.53)**
5	*rs7810357*	*KCND2*	G	442 (92.9%)	462 (96.2%)	**0.019**	1.00
			A	34 (7.1%)	18 (3.8%)		**0.50 (0.28**–**0.89)**
6	*rs7793037*	*KCND2*	A	247 (51.5%)	269 (56.5%)	0.096	1.00
			G	233 (48.5%)	207 (43.5%)		0.81 (0.62–1.04)
7	*rs2192373*	*KCND2*	C	448 (93.7%)	459 (94.8%)	0.400	1.00
			T	30 (6.3%)	25 (5.2%)		0.79 (0.46–1.37)
8	*rs802372*	*KCND2*	A	252 (52.9%)	242 (50.2%)	0.401	1.00
			G	224 (47.1%)	240 (49.8%)		1.12 (0.87–1.44)
9	*rs1527650*	*KCND2*	T	458 (96.2%)	466 (96.7%)	0.629	1.00
			G	18 (3.8%)	16 (3.3%)		0.84 (0.42–1.68)
10	*rs10278347*	*KCND2*	C	252 (52.9%)	226 (47.3%)	0.089	1.00
			A	224 (47.1%)	252 (52.7%)		1.25 (0.97–1.61)
11	*rs2402539*	*KCND2*	T	242 (51.1%)	235 (49.8%)	0.683	1.00
			C	232 (48.9%)	237 (50.2%)		1.06 (0.82–1.36)
12	*rs6979618*	*KCND2*	A	248 (51.7%)	282 (59.0%)	**0.024**	1.00
			G	232 (48.3%)	196 (41.0%)		**0.74 (0.58**–**0.96)**
13	*rs7779895*	*KCND2*	C	266 (58.3%)	250 (56.1%)	0.539	1.00
			A	190 (41.7%)	196 (43.9%)		1.09 (0.83–1.42)
14	*rs17142875*	*KCND2*	A	253 (52.5%)	252 (52.3%)	0.962	1.00
			G	229 (47.5%)	230 (47.7%)		1.01 (0.78–1.30)
15	*rs4727911*	*KCND2*	G	236 (50.2%)	252 (52.1%)	0.547	1.00
			T	234 (49.8%)	232 (47.9%)		0.93 (0.72–1.19)
16	*rs7795646*	*KCND2*	A	262 (54.8%)	252 (52.7%)	0.498	1.00
			G	216 (45.2%)	226 (47.3%)		1.09 (0.85–1.41)
17	*rs2896298*	*KCND2*	T	286 (59.1%)	273 (57.4%)	0.603	1.00
			C	198 (40.9%)	203 (42.6%)		1.07 (0.83–1.39)
18	*rs1072198*	*KCND2*	T	445 (93.1%)	439 (91.1%)	0.188	1.00
			C	33 (6.9%)	43 (8.9%)		1.38 (0.86–2.21)
19	*rs17142891*	*KCND2*	G	279 (58.4%)	269 (57.0%)	0.673	1.00
			A	199 (41.6%)	203 (43.0%)		1.06 (0.82–1.37)
20	*rs11983106*	*KCND2*	G	459 (96.0%)	464 (95.9%)	0.958	1.00
			T	19 (4.0%)	20 (4.1%)		1.02 (0.54–1.94)
21	*rs727228*	*KCND2*	T	281 (58.5%)	272 (56.2%)	0.431	1.00
			A	199 (41.5%)	212 (43.8%)		1.11 (0.86–1.43)

^#^*P* value and OR adjusted by age and sex; *p_Bonferroni_<0.005. The bold represent the statistically significant correlations (*p* < 0.05), or meaningful models or values.

Further, we conducted analyses examining whether ASD risk differed according to SNPs in five genetic inheritance models. As shown in Table 5, the genotype frequency of *rs477135* in *KCNB1* differed significantly between case and control groups (*p* < 0.05), but it was statistically insignificant after being adjusted by FDR-based correction. In *KCND2*, there were statistically significant differences in the genotype frequencies of *rs1990429*, *rs7800545*, *rs7793864*, *rs7810357*, and *rs6979618* between the two groups (*p* < 0.05). Notably, after the *p*-values were adjusted by FDR-based correction, *rs7800545* (OR = 0.49, 95% CI = 0.29–0.83, *p*_*FDR*_ = 0.045) in the dominant model and *rs1990429* (OR = 0.36, 95% CI = 0.22–0.60, *p*_*FDR*_ < 0.001) and *rs7793864* (OR = 0.15, 95% CI = 0.06–0.41, *p*_*FDR*_ < 0.001) in the over-dominant model were associated with a reduced risk of ASD (for further details see Supplementary Tables 6, 7).

**TABLE 5:** *KCNB1* and *KCND2* single nucleotide polymorphisms (SNPs) in different genetic models associated with autism spectrum disorder (ASD) risk.

SNPs ID	Gene	Model	Genotype	Control (243)	Case (243)	OR (95 CI)	*P*	AIC	BIC	*P*_*FDR*_
*rs477135*	*KCNB1*	Codominant	T/T	167 (70.2%)	147 (61.5%)	1.00	0.031	662.0	682.8	
			A/T	57 (23.9%)	83 (34.7%)	1.63 (1.09–2.44)				
			A/A	14 (5.9%)	9 (3.8%)	0.70 (0.29–1.68)				
		Dominant	T/T	167 (70.2%)	147 (61.5%)	1.00	0.059	663.4	680.0	
			A/T–A/A	71 (29.8%)	92 (38.5%)	1.44 (0.99–2.12)				
		Recessive	T/T–A/T	224 (94.1%)	230 (96.2%)	1.00	0.240	665.6	682.2	
			A/A	14 (5.9%)	9 (3.8%)	0.60 (0.26–1.43)				
		**Over-dominant**	T/T–A/A	181 (76%)	156 (65.3%)	1.00	**0.012**	**660.6**	**677.3**	0.336
			A/T	57 (23.9%)	83 (34.7%)	1.67 (1.12–2.49)				
		Log-additive	−	−	−	1.20 (0.87–1.64)	0.260	665.7	682.3	
*rs1990429*	*KCND2*	Codominant	G/G	180 (75.3%)	212 (87.6%)	1.00	<0.0001	**652.6**	673.4	
			G/A	59 (24.7%)	26 (10.7%)	0.37 (0.22–0.61)				
			A/A	0 (0%)	4 (1.6%)	NA (0.00–NA)				
		Dominant	G/G	180 (75.3%)	212 (87.6%)	1.00	<0.0001	659.3	676.0	
			G/A–A/A	59 (24.7%)	30 (12.4%)	0.42 (0.26–0.68)				
		Recessive	G/G–G/A	239 (100%)	238 (98.3%)	1.00	0.022	666.9	683.6	
			A/A	0 (0%)	4 (1.6%)	NA (0.00–NA)				
		**Over-dominant**	G/G–A/A	180 (75.3%)	216 (89.3%)	1.00	**<0.0001**	655.1	**671.8**	< 0.001*
			G/A	59 (24.7%)	26 (10.7%)	**0.36 (0.22–0.60)**				
		Log-additive	−	−	−	0.52 (0.33–0.81)	0.0035	663.6	680.3	
*rs7800545*	*KCND2*	Codominant	A/A	194 (80.5%)	214 (89.2%)	1.00	0.024	666.9	687.8	
			G/A	44 (18.3%)	24 (10%)	0.49 (0.28–0.83)				
			G/G	3 (1.2%)	2 (0.8%)	0.59 (0.10–3.58)				
		**Dominant**	A/A	194 (80.5%)	214 (89.2%)	1.00	0.0064	**665.0**	**681.7**	0.045*
			G/A–G/G	47 (19.5%)	26 (10.8%)	**0.49 (0.29–0.83)**				
		Recessive	A/A–G/A	238 (98.8%)	238 (99.2%)	1.00	0.640	672.2	688.9	
			G/G	3 (1.2%)	2 (0.8%)	0.65 (0.11–3.96)				
		Over-dominant	A/A–G/G	197 (81.7%)	216 (90%)	1.00	0.0076	665.3	682.0	
			G/A	44 (18.3%)	24 (10%)	0.49 (0.29–0.84)				
		Log-additive	−	−	−	0.54 (0.33–0.87)	0.0093	665.6	682.3	
*rs7793864*	*KCND2*	Codominant	T/T	213 (88.4%)	235 (97.5%)	1.00	<0.0001	654.7	675.5	
			A/T	28 (11.6%)	5 (2.1%)	0.16 (0.06–0.41)				
			A/A	0 (0%)	1 (0.4%)	NA (0.00–NA)				
		Dominant	T/T	213 (88.4%)	235 (97.5%)	1.00	<0.0001	656.3	673.0	
			A/T–A/A	28 (11.6%)	6 (2.5%)	0.19 (0.08–0.46)				
		Recessive	T/T–A/T	241 (100%)	240 (99.6%)	1.00	0.240	672.2	688.9	
			A/A	0 (0%)	1 (0.4%)	NA (0.00–NA)				
		**Over-dominant**	T/T–A/A	213 (88.4%)	236 (97.9%)	1.00	**<0.0001**	**653.9**	**670.6**	< 0.001*
			A/T	28 (11.6%)	5 (2.1%)	**0.15 (0.06–0.41)**				
		Log-additive	−	−	−	0.23 (0.10–0.54)	<0.0001	659.1	675.8	
*rs7810357*	*KCND2*	Codominant	G/G	205 (86.1%)	222 (92.5%)	1.00	0.040	663.0	683.9	
			G/A	32 (13.4%)	18 (7.5%)	0.51 (0.28–0.94)				
			A/A	1 (0.4%)	0 (0%)	0.00 (0.00–NA)				
		Dominant	G/G	205 (86.1%)	222 (92.5%)	1.00	0.019	662.0	678.6	
			G/A–A/A	33 (13.9%)	18 (7.5%)	0.49 (0.27–0.90)				
		Recessive	G/G–G/A	237 (99.6%)	240 (100%)	1.00	0.220	665.9	682.6	
			A/A	1 (0.4%)	0 (0%)	0.00 (0.00–NA)				
		Over-dominant	G/G–A/A	206 (86.5%)	222 (92.5%)	1.00	0.028	662.6	679.3	
			G/A	32 (13.4%)	18 (7.5%)	0.51 (0.28–0.94)				
		**Log-additive**	−	−	−	0.49 (0.27–0.88)	**0.015**	**661.5**	**678.2**	0.079
*rs6979618*	*KCND2*	Codominant	A/A	68 (28.3%)	82 (34.3%)	1.00	0.058	665.8	686.7	
			A/G	112 (46.7%)	118 (49.4%)	0.86 (0.57–1.30)				
			G/G	60 (25%)	39 (16.3%)	0.54 (0.32–0.91)				
		Dominant	A/A	68 (28.3%)	82 (34.3%)	1.00	0.150	667.4	684.1	
			A/G–G/G	172 (71.7%)	157 (65.7%)	0.75 (0.51–1.11)				
		**Recessive**	A/A–A/G	180 (75%)	200 (83.7%)	1.00	**0.023**	**664.3**	**681.0**	0.097
			G/G	60 (25%)	39 (16.3%)	0.59 (0.38–0.93)				
		Over-dominant	A/A–G/G	128 (53.3%)	121 (50.6%)	1.00	0.620	669.3	686.0	
			A/G	112 (46.7%)	118 (49.4%)	1.10 (0.76–1.57)				
		Log-additive	−	−	−	0.75 (0.58–0.97)	0.026	664.5	681.2	

OR, odds ratio; CI, confidence interval; AIC, akaike’ information criterion; BIC, bayesian information criterion; *p_FDR_*, FDR corrected *p* value. *p < 0.05. NA, not applicable. The bold represent the statistically significant correlations (*p* < 0.05), or meaningful models or values.

### 3.3. Haplotype analysis

**FIGURE 1:**
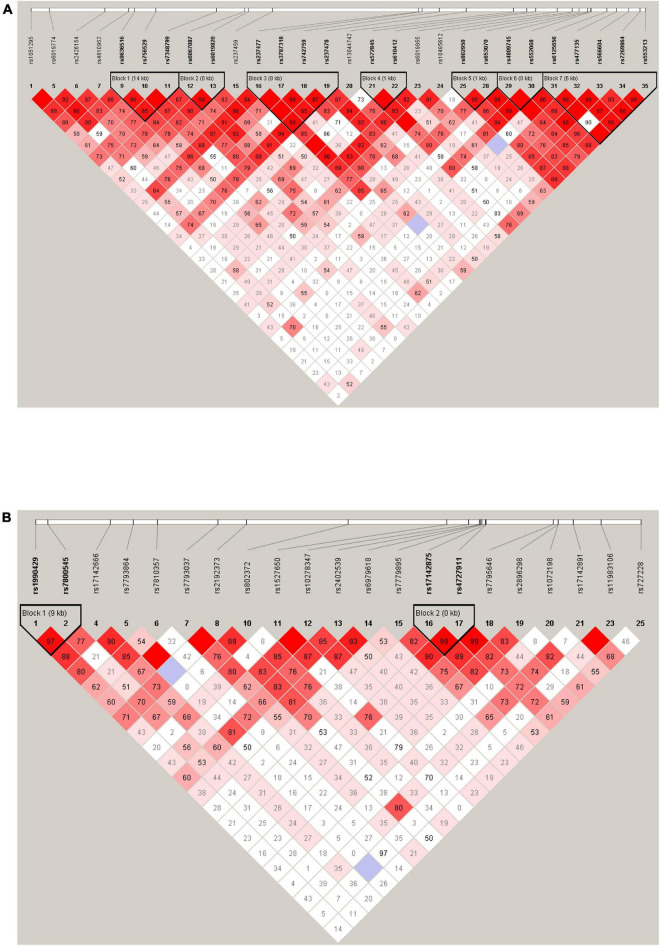
Haplotype block map for 28 tag SNPs of the KCNB1 gene **(A)**. Haplotype block map for 21 tag SNPs of the KCND2 gene **(B)**. Numbers in squares indicate D’ values.

The LD analysis revealed that among 28 tag SNPs in the *KCNB1* gene, 20 SNPs were in high LD (*r*^2^ ≥ 0.8) and were arranged in seven haplotype blocks with frequencies > 5%. Further details for all blocks are shown in Figure 1A. There were no significant differences in the haplotype frequencies between patients and controls (see Table 6).

**TABLE 6:** Distribution of *KCNB1* haplotypes in cases and controls adjusted by age and sex (frequency more than 5).

Block	No.	Haplotypes	Case ratio (243)	Control ratio (243)	*P*	OR (95% CI)
Block 1	1	AAT^c^	0.516	0.516		1.00
	2	GGT^c^	0.390	0.401	0.410	0.89 (0.68–1.17)
	3	GAC^c^	0.076	0.061	0.280	1.32 (0.79–2.21)
Block 2	1	AG^d^	0.496	0.507		1.00
	2	GA^d^	0.384	0.397	0.530	0.92 (0.70–1.20)
	3	GG^d^	0.118	0.094	0.190	1.32 (0.87–2.00)
Block 3	1	TTGC^e^	0.392	0.403		1.00
	2	CTGT^e^	0.312	0.320	0.630	1.08 (0.80–1.45)
	3	TTGT^e^	0.139	0.136	0.520	1.14 (0.77–1.69)
	4	TCAC^e^	0.088	0.072	0.150	1.45 (0.87–2.41)
	5	TTAC ^e^	0.060	0.066	0.890	1.04 (0.61–1.78)
Block 4	1	GA^f^	0.818	0.837		1.00
	2	AC^f^	0.133	0.128	0.740	0.94 (0.63–1.38)
Block 5	1	CC^g^	0.650	0.663		1.00
	2	CT^g^	0.180	0.162	0.350	1.18 (0.83–1.68)
	3	AT^g^	0.163	0.173	0.450	0.87 (0.61–1.25)
Block 6	1	GA^h^	0.537	0.561		1.00
	2	GC^h^	0.339	0.334	0.890	1.02 (0.77–1.35)
	3	AC^h^	0.125	0.105	0.240	1.28 (0.85–1.93)
Block 7	1	GTATA^i^	0.605	0.644		1.00
	2	GTATG^i^	0.188	0.180	0.980	0.99 (0.70–1.41)
	3	GAGCG^i^	0.114	0.093	0.230	1.29 (0.85–1.95)
	4	AAGTG^i^	0.075	0.068	0.330	1.28 (0.78–2.40)

^c^The order of SNPs in estimated analysis of haplotypes frequency: *rs9636516*, *rs756529*, and *rs7348799*; ^d^The order of SNPs in estimated analysis of haplotypes frequency: *rs6067087* and *rs6019820*; ^e^The order of SNPs in estimated analysis of haplotypes frequency: *rs237477*, *rs3787318*, *rs742759*, and *r237478*; ^f^The order of SNPs in estimated analysis of haplotypes frequency: *rs572845* and *rs610412*; ^g^The order of SNPs in estimated analysis of haplotypes frequency: *rs802950* and *rs653070*; ^h^The order of SNPs in estimated analysis of haplotypes frequency: *rs4809745* and *rs552068*; ^i^The order of SNPs in estimated analysis of haplotypes frequency: *rs6125656*, *rs477135*, *rs566604*, *rs7269864*, and *rs553213*.

There were four tag SNPs in high LD (*r*^2^ ≥ 0.8) arranged in two haplotype blocks with frequencies > 5% among 21 studied SNPs in the *KCND2* gene. Haplotype blocks were constructed by *rs1990429*/*rs7800545* and *rs17142875*/*rs4727911* (shown in Figure 1B). We found that the *rs1990429* A–*rs7800545* G haplotype carried a lower risk of ASD in Block 1. However, this was no longer statistically significant after Bonferroni correction. There were no statistically meaningful differences between the two groups in Block 2 (see Table 7).

**TABLE 7:** Distribution of *KCND2* haplotypes in cases and controls adjusted by age and sex (frequency more than 5).

Block	No.	Haplotypes	Case ratio (243)	Control ratio (243)	*P*	OR (95% CI)
Block 1	1	GA^j^	0.898	0.870	**0.026**	1.00
	2	AG^j^	0.077	0.099		0.58 (0.36–0.93)
Block 2	1	AT^k^	0.485	0.493	0.770	1.00
	2	GG^k^	0.475	0.477		1.04 (0.81–1.33)

^j^The order of SNPs in estimated analysis of haplotypes frequency: *rs1990429* and *rs7800545*; ^k^The order of SNPs in estimated analysis of haplotypes frequency: *rs17142875* and *rs4727911*. The bold represent the statistically significant correlations (*p* < 0.05), or meaningful models or values.

### 3.4. Association between genotype and phenotype in ASD

According to the above positive results, we selected three SNPs (*rs1990429*, *rs7800545*, and *rs7793864*) under the optimal genetic model, to evaluate the correlations between *KCND2* genotype and ASD symptom severity, including the ADI–R and ADOS domains. The analysis of variance revealed that the scores on the ADOS did not significantly differ between the two groups. The results showed that the G/A genotype of *rs1990429* in the over-dominant model and the G/A–G/G genotype of *rs7800545* in the dominant model had lower scores for the ADI–R RRB domain after adjusting for age, sex, and IQ (*p* = 0.001). However, no significant associations were observed for *rs7793864* in the over-dominant model (shown in Tables 8, 9).

**TABLE 8:** The genotype association of *KCND2* with ADOS–CSS adjusted by age, sex, and IQ.

SNPs	Genetic model	Genotype	SA–CSS	*F*	*P*	RRB–CSS	*F*	*P*	ADOS–CSS	*F*	*P*
*rs1990429*	Over-dominant	G/G–A/A	7.13 ± 1.66	0.351	0.554	6.44 ± 1.88	3.363	0.069	6.82 ± 1.59	0.054	0.817
		G/A	7.39 ± 1.61			5.56 ± 2.36			6.78 ± 1.56		
*rs7800545*	Dominant	A/A	7.15 ± 1.67	0.080	0.777	6.41 ± 1.89	1.785	0.183	6.82 ± 1.59	0.410	0.523
		G/A–G/G	7.00 ± 1.75			5.72 ± 2.34			6.56 ± 1.69		
*rs7793864*	Over-dominant	T/T–A/A	7.13 ± 1.69	1.724	0.191	6.35 ± 1.95	0.434	0.511	6.79 ± 1.61	0.536	0.465
		A/T	8.00 ± 0.82			5.50 ± 1.92			7.25 ± 1.26		

ADOS, autism diagnostic observation schedule; SA, social affect; RRB, restricted repetitive behavior; CSS, calibrated severity scores.

**TABLE 9:** The genotype association of *KCND2* with ADI–R adjusted by age, sex, and IQ.

SNPs	Genetic model	Genotype	SOC	*F*	*P*	VC	*F*	*P*	NVC	*F*	*P*	*RRB*	*F*	*P*
*rs1990429*	Over-dominant	G/G–A/A	22.88 ± 4.50	0.042	0.838	17.33 ± 3.64	0.022	00.883	10.98 ± 2.81	0.611	0.436	5.83 ± 2.78	10.95	**0.001**
		G/A	22.33 ± 6.42			17.09 ± 4.30			11.50 ± 2.90			3.55 ± 2.33		
*rs7800545*	Dominant	A/A	22.96 ± 4.55	1.115	0.293	17.38 ± 3.64	0.007	0.933	11.05 ± 2.80	0.752	0.387	5.81 ± 2.79	12.567	**0.001**
		G/A–G/G	21.67 ± 6.22			17.33 ± 4.36			11.61 ± 2.79			3.50 ± 2.26		
*rs7793864*	Over-dominant	T/T–A/A	22.78 ± 4.68	0.851	0.358	17.36 ± 3.71	0.392	0.533	11.10 ± 2.80	0.410	0.523	5.58 ± 2.84	0.031	0.860
		A/T	23.25 ± 8.26			15.00 ± 4.24			9.75 ± 3.50			4.50 ± 2.65		

ADI–R, autism diagnostic interview–revised; ADI–R SOC, ADI–R social; ADI–R VC, ADI–R communication for verbal; ADI–R NVC, ADI–R communication for non-verbal; ADI–R RRB, ADI–R restricted repetitive behaviors. The bold represent the statistically significant correlations (*p* < 0.05), or meaningful models or values.

## 4. Discussion

In this case-control study, we aimed to assess the linkage of polymorphisms of the *KCNB1* and *KCND2* genes (including tag SNPs and pathogenic SNPs) with the risk of ASD using allele frequencies, genotype frequencies, and haplotype analyses. We also explored the potential relationship between genetic polymorphism and ASD symptom severity. This study found that *KCND2 rs1990429*, *rs7800545*, and *rs7793864* were associated with ASD risk, of which *rs1990429* and *rs7800545* were highly correlated with repetitive stereotyped behavior in ASD.

Our study did not find *KCNB1* to be associated with ASD risk. Previous research has found that the V378A variant in *KCNB1* influenced the expression and localization of Kv2.1 protein, thereby perturbing the voltage-activated current, the ionic selectivity, and the ability of the channel to repolarize (19). There was evidence that mutated *KCNB1* caused “autism-like” features, including repetitive behaviors and impulsivity, and seizure susceptibility, by affecting the highly conserved structural function of Kv2.1 (20). Individuals with *KCNB1* pathogenic variants (such as p.Glu43Gly, p.Arg312His, and p.Trp369Arg) were at high risk of ASD and exhibited impaired communication and socialization (21). In particular, the missense variants in *KCNB1*, including *rs587777848* (S347R), *rs587777849* (T374I), and *rs587777850* (G379R), have been shown to be related to language and motor delays (similar to autistic behaviors). These three missense variants were located in the functionally important pore domain of the Kv2.1 protein and produced a loss-of-function effect, resulting in altered ion selectivity and reduced current density at depolarized voltages (18). Regrettably, we did not find that *rs587777848*, *rs587777849*, and *rs587777850* were polymorphic loci or find any significant associations between mutations in *KCNB1* and ASD in the Chinese Han population. There are several possible explanations for the apparent differences between our findings and prior studies. ASD is a disorder of great genetic complexity and heterogeneity, making it difficult to delineate the contribution of any single gene to the risk of this disease, where a single candidate gene might be accountable for various disease phenotypes. In addition, differences in the characteristics of participants may be the main reason for the different results. In previous research, patients had comorbid epilepsy, intellectual disability, or developmental delay, while our patients were confined to those with single-incidence ASD among the Chinese Han population. This implies that mutation in *KCNB1* may be an etiological factor shared by epilepsy, intellectual disability, and ASD, rather than the specific pathogenic factor in ASD.

*KCND2* is located on chromosome 7 (7q31.31). A clinical case report confirmed that genomic copy number loss in this region mediated the core clinical features of ASD, such as stereotypic movements, impairment of social interaction, and poor social skills (22, 23). This means that *KCND2* is located on the most significant susceptibility locus in autism. A study has shown that Kv4.2 protein expression was downregulated in the hippocampal of Fmr1 knockout mice (a common model of ASD), resulting in excess neuronal excitability (24). *KCND2* knockout mice exhibited delayed synaptic maturation and hippocampal-dependent learning and memory deficits, which suggested a critical role of Kv4.2 in cognition (25, 26). In addition, Mikhailov et al. (27) identified three substituted *KCND2* variants (N544S, F538S, and R539L) in patients with ASD, which interfered with the expression of Kv4.2 protein. Recent research has also indicated that *KCND2 rs10239799* allele C, which was positively selected as an ASD risk allele, was essential in impacting higher-order brain functions such as cognition, behavior, and memory (28). Lee et al. (29) found that the deleterious *de novo* variant V404M in *KCND2* impaired potassium channel inactivation in monozygotic twins with ASD and epilepsy. *In vitro* studies found that the substitution variant of V404M is located in S6 transmembrane segment, which surrounds the central ion conduction pathway mediating closed state inactivation (30). In the Kv4.2 channel with V404M mutant, the inactivation is enhanced directly from preopen closed states, while the pore closure rate is dramatically slowed when the channel opens compared to Kv4.2 WT (12, 31). However, the pathogenic mutations in *KCND2* (N544S, F538S, R539L, and V404M), which have been confirmed to be present in ASD in previous studies, were neither found in the dbSNP database nor detected by MassArray sequencing in this study. Overall, we and others have reported similar findings that *KCND2* was crucial for the occurrence of ASD. This is the first time that we found the G/A genotype of *rs1990429*, G/A–G/G genotype of *rs7800545*, and A/T genotype of *rs7793864* reduced the risk of ASD. To date, there are lack of studies related to biological functionality of these three gene loci. Further research will be required to explore the effects of *rs1990429*, *rs7800545*, and *rs7793864* in *KCND2* on Kv4.2 channel expression and function, and their impact on ASD at the cellular levels and animal models.

To improve the detection capability, haplotype analyses were conducted to estimate the combined influence of genetic variants on ASD risk. Regrettably, although we derived 7 haplotype blocks across 28 SNPs in *KCNB1*, they did not play a role in ASD. Of the two haplotype blocks detected in *KCND2*, the AG (*rs1990429*–*rs7800545*) was a protective factor for ASD, while this statistical difference disappeared after correction for multiple comparisons. In addition, we used ADOS and ADI–R to evaluate the core symptoms of ASD. We found that patients with the G/A genotype of *rs1990429* and the G/A–G/G genotype of *rs7800545* had lower severity of stereotyped behaviors. Connolly et al. (32) pointed out that *KCND2* was significantly associated with overly serious facial expressions in patients with ASD, which is part of the communication subscale of the Social Responsiveness Scale. However, there is evidence to confirm that reduced Kv4.2 expression altered dendritic spine morphology and density, but did not induce perseverative or repetitive behavior in mice (33). In general, there is a lack of consistent association between *KCND2* and ASD phenotype.

This study has several limitations that may influence the interpretation of the study results. First, the best model was selected based on the smallest AIC and BIC, which were estimated by maximum likelihood estimation. Given the principle of parsimony, this approach increases the risk of Type I errors. Moreover, this strategy yielded a specific but not very sensitive rule for model selection. Thus, it is necessary to explore the relationship between the risk genotype and the incidence of ASD in multiple models. Second, the results might be influenced by other confounding factors, such as early exposure factors, physical and chemical factors, and nutritional imbalance. Third, our study focused on the Chinese Han population, so further research should include a multi-ethnic population. Finally, studies of the altered function of mutated genes should be validated in animal models.

## 5. Conclusion

Given its role in synaptic function and plasticity, we examined the role of Kv channels in the ASD risk. Our results support *KCND2* as a susceptibility gene for ASD and illustrate that three SNPs (*rs1990429*, *rs7800545*, and *rs7793864*) in the *KCND2* gene reduce the risk of developing ASD, among which *rs1990429* and *rs7800545* alleviate the severity of RRB. These findings will provide important insights into ASD etiopathogenesis and genetic etiology.

## Data availability statement

The datasets presented in this study can be found in dbSNP 156 database with ID 1063426 (https://www.ncbi.nlm.nih.gov/SNP/snp_viewBatch.cgi?sbid=1063426).

## Ethics statement

The studies involving human participants were reviewed and approved by the Institutional Review Board of Harbin Medical University for Medical Sciences. Written informed consent to participate in this study was provided by the participants’ legal guardian/next of kin.

## Author contributions

MZ provided financial support for the conduct of the research and preparation of the manuscript. MZ and CS designed the study. WX, PG, and FW performed the clinical assessment and experiments. ZL and XY collected the data. MZ and YC analyzed the data. ZL wrote the manuscript. All authors read, revised, and approved the final manuscript.

## Footnotes

1
http://bioinfo.iconcologia.net/SNPstats

## References

[1] Maenner MJ Shaw KA Bakian AV Bilder DA Durkin MS Esler A Prevalence and characteristics of autism spectrum disorder among children aged 8 years – autism and developmental disabilities monitoring network, 11 sites, united states, 2018. *MMWR Surveill Summ.* (2021) 70:1–16. 10.15585/mmwr.ss7011a1 34855725 PMC8639024

[2] Zhou H Xu X Yan W Zou X Wu L Luo X Prevalence of autism spectrum disorder in china: a nationwide multi-center population-based study among children aged 6 to 12 years. *Neurosci Bull.* (2020) 36:961–71. 10.1007/s12264-020-00530-6 32607739 PMC7475160

[3] Sandin S Lichtenstein P Kuja-Halkola R Hultman C Larsson H Reichenberg A. The heritability of autism spectrum disorder. *Jama.* (2017) 318:1182–4. 10.1001/jama.2017.12141 28973605 PMC5818813

[4] Huang Y Zhao Y Ren Y Yi Y Li X Gao Z Identifying genomic variations in monozygotic twins discordant for autism spectrum disorder using whole-genome sequencing. *Mol Ther Nucleic Acids.* (2019) 14:204–11. 10.1016/j.omtn.2018.11.015 30623854 PMC6325071

[5] Cheng P Qiu Z Du Y. Potassium channels and autism spectrum disorder: an overview. *Int J Dev Neurosci.* (2021) 81:479–91. 10.1002/jdn.10123 34008235

[6] Wang J Feng S Li M Liu Y Yan J Tang Y Increased expression of Kv10.2 in the hippocampus attenuates valproic acid-induced autism-like behaviors in rats. *Neurochem Res.* (2019) 44:2796–808. 10.1007/s11064-019-02903-4 31728858

[7] Bee S Ringland A Coutellier L. Social impairments in mice lacking the voltage-gated potassium channel Kv3.1. *Behav Brain Res.* (2021) 413:113468. 10.1016/j.bbr.2021.113468 34274375

[8] Gamal El-Din TM Lantin T Tschumi CW Juarez B Quinlan M Hayano JH Autism-associated mutations in Kv7 channels induce gating pore current. *Proc Natl Acad Sci USA.* (2021) 118:105092. 10.1073/pnas.2112666118 34728568 PMC8609342

[9] Speca DJ Ogata G Mandikian D Bishop HI Wiler SW Eum K Deletion of the Kv2.1 delayed rectifier potassium channel leads to neuronal and behavioral hyperexcitability. *Genes Brain Behav.* (2014) 13:394–408. 10.1111/gbb.12120 24494598 PMC4077602

[10] Lee HY Ge WP Huang W He Y Wang GX Rowson-Baldwin A Bidirectional regulation of dendritic voltage-gated potassium channels by the fragile X mental retardation protein. *Neuron.* (2011) 72:630–42. 10.1016/j.neuron.2011.09.033 22099464 PMC3433402

[11] Kang SK Vanoye CG Misra SN Echevarria DM Calhoun JD O’Connor JB Spectrum of Kv 2.1 dysfunction in Kcnb1-associated neurodevelopmental disorders. *Ann Neurol.* (2019) 86:899–912. 10.1002/ana.25607 31600826 PMC7025436

[12] Zhang Y Tachtsidis G Schob C Koko M Hedrich UBS Lerche H Kcnd2 variants associated with global developmental delay differentially impair Kv4.2 channel gating. *Hum Mol Genet.* (2021) 30:2300–14. 10.1093/hmg/ddab192 34245260 PMC8600029

[13] Keller R Basta R Salerno L Elia M. Autism, epilepsy, and synaptopathies: a not rare association. *Neurol Sci.* (2017) 38:1353–61. 10.1007/s10072-017-2974-x 28455770

[14] Bar C Kuchenbuch M Barcia G Schneider A Jennesson M Le Guyader G Developmental and epilepsy spectrum of Kcnb1 encephalopathy with long-term outcome. *Epilepsia.* (2020) 61:2461–73. 10.1111/epi.16679 32954514

[15] Gotham K Pickles A Lord C. Standardizing ados scores for a measure of severity in autism spectrum disorders. *J Autism Dev Disord.* (2009) 39:693–705. 10.1007/s10803-008-0674-3 19082876 PMC2922918

[16] Gotham K Risi S Pickles A Lord C. The autism diagnostic observation schedule: revised algorithms for improved diagnostic validity. *J Autism Dev Disord.* (2007) 37:613–27. 10.1007/s10803-006-0280-1 17180459

[17] Hus V Gotham K Lord C. Standardizing ados domain scores: separating severity of social affect and restricted and repetitive behaviors. *J Autism Dev Disord.* (2014) 44:2400–12. 10.1007/s10803-012-1719-1 23143131 PMC3612387

[18] Torkamani A Bersell K Jorge BS Bjork RL Jr. Friedman JR Bloss CS De Novo Kcnb1 mutations in epileptic encephalopathy. *Ann Neurol.* (2014) 76:529–40. 10.1002/ana.24263 25164438 PMC4192091

[19] Thiffault I Speca DJ Austin DC Cobb MM Eum KS Safina NP A novel epileptic encephalopathy mutation in kcnb1 disrupts Kv2.1 ion selectivity, expression, and localization. *J Gen Physiol.* (2015) 146:399–410. 10.1085/jgp.201511444 26503721 PMC4621747

[20] Hawkins NA Misra SN Jurado M Kang SK Vierra NC Nguyen K Epilepsy and neurobehavioral abnormalities in mice with a dominant-negative Kcnb1 pathogenic variant. *Neurobiol Dis.* (2021) 147:105141. 10.1016/j.nbd.2020.105141 33132203 PMC7725922

[21] Bar C Breuillard D Kuchenbuch M Jennesson M Le Guyader G Isnard H Adaptive behavior and psychiatric comorbidities in Kcnb1 encephalopathy. *Epilepsy Behav.* (2022) 126:108471. 10.1016/j.yebeh.2021.108471 34915430

[22] Bailey A Hervas A Matthews N Palferman S Wallace S Aubin A A full genome screen for autism with evidence for linkage to a region on chromosome 7q. *Hum Mol Genet*. (1998) 7:571–8. 10.1093/hmg/7.3.571 9546821

[23] Okamoto N Hatsukawa Y Shimojima K Yamamoto T. Submicroscopic deletion in 7q31 encompassing Cadps2 and Tspan12 in a child with autism spectrum disorder and Phpv. *Am J Med Genet A.* (2011) 155A:1568–73. 10.1002/ajmg.a.34028 21626674

[24] Gross C Yao X Pong DL Jeromin A Bassell GJ. Fragile X mental retardation protein regulates protein expression and mrna translation of the potassium channel Kv4.2. *J Neurosci.* (2011) 31:5693–8. 10.1523/JNEUROSCI.6661-10.2011 21490210 PMC3089949

[25] Lugo JN Brewster AL Spencer CM Anderson AE. Kv4.2 knockout mice have hippocampal-dependent learning and memory deficits. *Learn Mem.* (2012) 19:182–9. 10.1101/lm.023614.111 22505720 PMC3348517

[26] Kim E Hoffman DA. Dynamic regulation of synaptic maturation state by voltage-gated a-type K+ channels in Ca1 hippocampal pyramidal neurons. *J Neurosci.* (2012) 32:14427–32. 10.1523/JNEUROSCI.2373-12.2012 23055512 PMC3489019

[27] Mikhailov A Choufani S Skaug J Kolozsvari D Marshall C Scherer S (editors) “Chromosomal translocation T (5; 7)(Q14; Q31) and missense mutations implicate the voltage-gated potassium channel Kv4. 2 Gene, Kcnd2, on 7q31 in autism,” in *Proceedings of the Annual Meeting of the American Society of Human Genetics*, Philadelphia, PA (2008).

[28] Prakash A Banerjee M. Genomic selection signatures in autism spectrum disorder identifies cognitive genomic tradeoff and its relevance in paradoxical phenotypes of deficits versus potentialities. *Sci Rep.* (2021) 11:10245. 10.1038/s41598-021-89798-w 33986442 PMC8119484

[29] Lee H Lin MC Kornblum HI Papazian DM Nelson SF. Exome sequencing identifies de novo gain of function missense mutation in Kcnd2 in identical twins with autism and seizures that slows potassium channel inactivation. *Hum Mol Genet.* (2014) 23:3481–9. 10.1093/hmg/ddu056 24501278 PMC4049306

[30] Swartz KJ. Towards a structural view of gating in potassium channels. *Nat Rev Neurosci.* (2004) 5:905–16. 10.1038/nrn1559 15550946

[31] Lin MA Cannon SC Papazian DM. Kv4.2 autism and epilepsy mutation enhances inactivation of closed channels but impairs access to inactivated state after opening. *Proc Natl Acad Sci USA.* (2018) 115:E3559–68. 10.1073/pnas.1717082115 29581270 PMC5899440

[32] Connolly JJ Glessner JT Hakonarson HA. Genome-wide association study of autism incorporating autism diagnostic interview-revised, autism diagnostic observation schedule, and social responsiveness scale. *Child Dev.* (2013) 84:17–33. 10.1111/j.1467-8624.2012.01838.x 22935194

[33] Tiwari D Schaefer TL Schroeder-Carter LM Krzeski JC Bunk AT Parkins EV The potassium channel Kv4.2 regulates dendritic spine morphology, electroencephalographic characteristics and seizure susceptibility in mice. *Exp Neurol.* (2020) 334:113437. 10.1016/j.expneurol.2020.113437 32822706 PMC7642025

