# Supplementary material for: The association between gene polymorphisms in voltage-gated potassium channels Kv2.1 and Kv4.2 and susceptibility to autism spectrum disorder
Source: Front Psychiatry. 2023 Jan 23;13:994166. doi: 10.3389/fpsyt.2022.994166 (PMC9900626; doi:10.3389/fpsyt.2022.994166)
Supplement: Supplementary file 1 [file Table_1.DOCX]

**Supplementary Table 1** The results of ADI-R and ADOS (±SD)

| **Variants** | **n** | **mean±SD** |
| --- | --- | --- |
| ADI-R |  |  |
| ADI- social | 162 | 22.79±4.73 |
| ADI-R comm-V | 122 | 17.34±3.69 |
| ADI-R comm-NV | 152 | 11.06±2.82 |
| ADI-R RRB | 162 | 5.56±2.81 |
| ADOS |  |  |
| SA-CSS | 166 | 7.14±1.67 |
| RRB-CSS | 166 | 6.34±1.94 |
| ADOS-CSS | 166 | 6.80±1.59 |

ADI-R = Autism Diagnostic Interview-Revised, ADI-R comm-V, ADI-R communication for verbal; ADI-R comm-NV, ADI-R communication for nonverbal; ADI-R RRB, ADI-R restricted repetitive behaviors, ADOS = Autism Diagnostic Observation Schedule, SA = social affect, RRB = restricted repetitive behavior, CSS = calibrated severity scores.

**Supplementary Table 2** Basic information of *KCNB1* SNPs

|  | **SNP ID** | **Gene** | **Genic position** | **Alleles^#^** | **MAF ^#^(CHB)** |
| --- | --- | --- | --- | --- | --- |
| 1 | *rs1051295* | *KCNB1* | intron variant, utr variant 3 prime | C/T | 0.459 |
| 2 | *rs6019774* | *KCNB1* | intron | C/T | 0.072 |
| 3 | *rs2426154* | *KCNB1* | intron | C/T | 0.418 |
| 4 | *rs4810952* | *KCNB1* | intron | C/T | 0.425 |
| 5 | *rs1961192* | *KCNB1* | intron | C/T | 0.219 |
| 6 | *rs9636516* | *KCNB1* | intron | A/G | 0.469 |
| 7 | *rs756529* | *KCNB1* | intron | A/G | 0.405 |
| 8 | *rs7348799* | *KCNB1* | intron | C/T | 0.070 |
| 9 | *rs6067087* | *KCNB1* | intron | A/G | 0.497 |
| 10 | *rs6019820* | *KCNB1* | intron | A/G | 0.392 |
| 11 | *rs237454* | *KCNB1* | intron | C/T | 0.386 |
| 12 | *rs237459* | *KCNB1* | intron | A/G | 0.443 |
| 13 | *rs237477* | *KCNB1* | intron | C/T | 0.320 |
| 14 | *rs3787318* | *KCNB1* | intron | C/T | 0.083 |
| 15 | *rs742759* | *KCNB1* | intron | A/G | 0.145 |
| 16 | *rs237478* | *KCNB1* | intron | C/T | 0.456 |
| 17 | *rs13044742* | *KCNB1* | intron | A/T | 0.111 |
| 18 | *rs572845* | *KCNB1* | intron | C/T | 0.130 |
| 19 | *rs610412* | *KCNB1* | intron | A/C | 0.173 |
| 20 | *rs6019855* | *KCNB1* | intron | G/T | 0.280 |
| 21 | *rs10485612* | *KCNB1* | intron | A/G | 0.083 |
| 22 | *rs802950* | *KCNB1* | intron | A/C | 0.173 |
| 23 | *rs490840* | *KCNB1* | intron | G/T | 0.288 |
| 24 | *rs802952* | *KCNB1* | intron | C/T | 0.197 |
| 25 | *rs653070* | *KCNB1* | intron | C/T | 0.340 |
| 26 | *rs4809745* | *KCNB1* | intron | A/G | 0.115 |
| 27 | *rs552068* | *KCNB1* | intron | A/C | 0.451 |
| 28 | *rs6125656* | *KCNB1* | intron | A/G | 0.072 |
| 29 | *rs477135* | *KCNB1* | intron | A/T | 0.189 |
| 30 | *rs566604* | *KCNB1* | intron | A/G | 0.179 |
| 31 | *rs7269864* | *KCNB1* | intron | C/T | 0.117 |
| 32 | *rs553213* | *KCNB1* | intron | C/T | 0.373 |
| 33 | *rs587777850* | *KCNB1* | intron variant, missense | C/T | 0.001 |
| 34 | *rs587777849* | *KCNB1* | intron variant, missense | A/G | - |
| 35 | *rs587777848* | *KCNB1* | intron variant, missense | G/T | - |

^#^refers to Chinese Han calculations based on Hapmap data; MAF, minor allele frequency.

**Supplementary Table 3** Basic information of *KCND2* SNPs

|  | **SNP ID** | **Gene** | **Genic position** | **Alleles^#^** | **MAF ^#^(CHB)** |
| --- | --- | --- | --- | --- | --- |
| 1 | *rs1990429* | *KCND2* | intron variant,utr variant 3 prime | A/G | 0.111 |
| 2 | *rs7800545* | *KCND2* | intron | A/G | 0.089 |
| 3 | *rs2191736* | *KCND2* | intron | A/G | 0.179 |
| 4 | *rs17142666* | *KCND2* | intron | A/G | 0.339 |
| 5 | *rs7793864* | *KCND2* | intron | A/T | 0.048 |
| 6 | *rs7810357* | *KCND2* | intron | A/G | 0.063 |
| 7 | *rs7793037* | *KCND2* | intron | A/G | 0.468 |
| 8 | *rs2192373* | *KCND2* | intron | C/T | 0.058 |
| 9 | *rs802359* | *KCND2* | intron | A/T | 0.470 |
| 10 | *rs802372* | *KCND2* | intron | C/T | 0.481 |
| 11 | *rs1527650* | *KCND2* | intron | G/T | 0.035 |
| 12 | *rs10278347* | *KCND2* | intron | A/C | 0.491 |
| 13 | *rs2402539* | *KCND2* | intron | C/T | 0.489 |
| 14 | *rs6979618* | *KCND2* | intron | A/G | 0.454 |
| 15 | *rs7779895* | *KCND2* | intron | A/C | 0.425 |
| 16 | *rs17142875* | *KCND2* | intron | A/G | 0.475 |
| 17 | *rs4727911* | *KCND2* | intron | G/T | 0.491 |
| 18 | *rs7795646* | *KCND2* | intron | A/G | 0.459 |
| 19 | *rs2896298* | *KCND2* | intron | C/T | 0.414 |
| 20 | *rs1072198* | *KCND2* | intron | A/G | 0.081 |
| 21 | *rs17142891* | *KCND2* | intron | A/G | 0.418 |
| 22 | *rs2189977* | *KCND2* | intron | A/C | 0.345 |
| 23 | *rs11983106* | *KCND2* | intron | G/T | 0.042 |
| 24 | *rs12673992* | *KCND2* | intron | A/G | 0.485 |
| 25 | *rs727228* | *KCND2* | utr variant 3 prime | A/T | 0.429 |

^#^refers to Chinese Han calculations based on Hapmap data; MAF, minor allele frequency.

**Supplementary Table 4** The primers used in the screening of SNPs in *KCNB1*

|  | **SNPs ID** |  | **Primers** | **Amplicon size (bp)** |
| --- | --- | --- | --- | --- |
| 1 | *rs1051295* | Forward | ACGTTGGATGCCAGCGCAAAACCCTTACTC | 109 |
|  |  | Reverse | ACGTTGGATGACTCACCCTGATGGTATCTC |  |
| 2 | *rs6019774* | Forward | ACGTTGGATGTGAACAGATGCGGACACTTG | 106 |
|  |  | Reverse | ACGTTGGATGCATGTTCTTGGGTCACACAG |  |
| 3 | *rs2426154* | Forward | ACGTTGGATGTTTGCAATAGTGGGCCAGAG | 114 |
|  |  | Reverse | ACGTTGGATGTGTTCCATTCTCTCCACCTG |  |
| 4 | *rs4810952* | Forward | ACGTTGGATGTGGCACATCCCCACATGAAT | 118 |
|  |  | Reverse | ACGTTGGATGCCTCATCTATAAAAAGGGAC |  |
| 5 | *rs1961192* | Forward | ACGTTGGATGTTAGGCCCTGGTAATACTGC | 119 |
|  |  | Reverse | ACGTTGGATGTATTGTTCACTGTGTGCCCC |  |
| 6 | *rs9636516* | Forward | ACGTTGGATGTTGAAGATAACACCGCCCAG | 100 |
|  |  | Reverse | ACGTTGGATGCCGACTCCCATCTTATTGAC |  |
| 7 | *rs756529* | Forward | ACGTTGGATGTCTGGCAGGAGCAACCAAAG | 89 |
|  |  | Reverse | ACGTTGGATGCAAATGCTGACATCTGACCG |  |
| 8 | *rs7348799* | Forward | ACGTTGGATGGGCTAAATCCACGGTGATTG | 118 |
|  |  | Reverse | ACGTTGGATGCACAAATGGCCTGTTTCTGG |  |
| 9 | *rs6067087* | Forward | ACGTTGGATGGAAACATGCCAGCAGCTATG | 100 |
|  |  | Reverse | ACGTTGGATGCCAGGAGTTCACATCTTTAC |  |
| 10 | *rs6019820* | Forward | ACGTTGGATGGGTGCTTAATAGATGCAGCC | 103 |
|  |  | Reverse | ACGTTGGATGCCCTGGTTTAGAGAGCATTG |  |
| 11 | *rs237454* | Forward | ACGTTGGATGAACTGTAAGTGCAAAGGCCC | 117 |
|  |  | Reverse | ACGTTGGATGATCCACCAGCTGAGCTAAAC |  |
| 12 | *rs237459* | Forward | ACGTTGGATGTATTACTCTGGTCCCCAGTC | 111 |
|  |  | Reverse | ACGTTGGATGAGCAACCACAGTTTGAGCAC |  |
| 13 | *rs237477* | Forward | ACGTTGGATGTACCTGAGAAGAGCCTTTGG | 105 |
|  |  | Reverse | ACGTTGGATGAAGGCCATGACAGACCTGAG |  |
| 14 | *rs3787318* | Forward | ACGTTGGATGACATCCCAGAATGCACAAGG | 103 |
|  |  | Reverse | ACGTTGGATGACCGAGCATTTCTCAACCTC |  |
| 15 | *rs742759* | Forward | ACGTTGGATGAGTCTGTGATCAGAGTCAGG | 118 |
|  |  | Reverse | ACGTTGGATGTTCCTTCATAACCCTGGTCC |  |
| 16 | *rs237478* | Forward | ACGTTGGATGGCTGCCAGTTCGAGTTTAAT | 119 |
|  |  | Reverse | ACGTTGGATGTTCTGGCAAACTGTATGGAG |  |
| 17 | *rs13044742* | Forward | ACGTTGGATGGGATTTGCTTAATAGTCTTC | 120 |
|  |  | Reverse | ACGTTGGATGGCAAATGGCACCAGTTCTTC |  |
| 18 | *rs572845* | Forward | ACGTTGGATGCTCCCTCATAAAGAACCTCC | 103 |
|  |  | Reverse | ACGTTGGATGTCCCCTTGGTCACTGTTTTG |  |
| 19 | *rs610412* | Forward | ACGTTGGATGGTCCCAGGTTGGAACTAATG | 99 |
|  |  | Reverse | ACGTTGGATGTCTGATCATGAAGACTGCCG |  |
| 20 | *rs6019855* | Forward | ACGTTGGATGCGAATGAAGTACCTGTGCTC | 99 |
|  |  | Reverse | ACGTTGGATGAGCTACTGGAAAGACAGGTG |  |
| 21 | *rs10485612* | Forward | ACGTTGGATGCCTCTCTGTAAATGGAAGGC | 93 |
|  |  | Reverse | ACGTTGGATGTGCTCCTTCCACCTTTCTTG |  |
| 22 | *rs802950* | Forward | ACGTTGGATGAAGTAGAAAGGGAGGGAGAC | 99 |
|  |  | Reverse | ACGTTGGATGGCCTTAGCACTGCTTTTCTC |  |
| 23 | *rs490840* | Forward | ACGTTGGATGCAAAGTGCTGGGATTACAGG | 100 |
|  |  | Reverse | ACGTTGGATGCAGAACTTTCTCCACAGTGC |  |
| 24 | *rs802952* | Forward | ACGTTGGATGACAGAGCAAGACTCTGTCTC | 102 |
|  |  | Reverse | ACGTTGGATGTACTGAAGTGTTTATGGACG |  |
| 25 | *rs653070* | Forward | ACGTTGGATGTTCCTTTCTCTCTCTCCCAC | 100 |
|  |  | Reverse | ACGTTGGATGCTGAAGACACAACAGTGGAC |  |
| 26 | *rs4809745* | Forward | ACGTTGGATGGTGAGTTGCTGTGTGTGGAG | 97 |
|  |  | Reverse | ACGTTGGATGCATTGCAGGAGCCTCTAGTC |  |
| 27 | *rs552068* | Forward | ACGTTGGATGTTCCTCTTCCACTCAGGACG | 108 |
|  |  | Reverse | ACGTTGGATGGAAGTGGCCACAACCTAGAC |  |
| 28 | *rs6125656* | Forward | ACGTTGGATGAGGAGTGTTCTTAGCTCTGG | 96 |
|  |  | Reverse | ACGTTGGATGATCTGTCTCTCCCATAGTCC |  |
| 29 | *rs477135* | Forward | ACGTTGGATGAAACCTACTCCACAGGGTTG | 100 |
|  |  | Reverse | ACGTTGGATGTGGGATTTCTCAGGCACAGC |  |
| 30 | *rs566604* | Forward | ACGTTGGATGTCAATGATGCATCTTCCTCC | 103 |
|  |  | Reverse | ACGTTGGATGTGCCAGTTAGGGATGCTTTG |  |
| 31 | *rs7269864* | Forward | ACGTTGGATGTTGTGACCTCTCCATGATCC | 105 |
|  |  | Reverse | ACGTTGGATGGCTCTGGCTACAGAAAATCC |  |
| 32 | *rs553213* | Forward | ACGTTGGATGTTGTCATTTGCTCCCAGCAC | 119 |
|  |  | Reverse | ACGTTGGATGAGCAACTTCTCTACGTCAGG |  |
| 33 | *rs587777850* | Forward | ACGTTGGATGCAATTTTCCCCAGGAGAGTC | 113 |
|  |  | Reverse | ACGTTGGATGAAAGCATCCCAGCCTCTTTC |  |
| 34 | *rs587777849* | Forward | ACGTTGGATGGATGTCTCCATACCCAACAG | 106 |
|  |  | Reverse | ACGTTGGATGATGAGGACGACACCAAGTTC |  |
| 35 | *rs587777848* | Forward | ACGTTGGATGTTGCTCATCCTCTTCCTTGC | 106 |
|  |  | Reverse | ACGTTGGATGAACTTGGTGTCGTCCTCATC |  |

**Supplementary Table 5** The primers used in the screening of SNPs in *KCND2*

|  | **SNPs ID** |  | **Primers** | **Amplicon size (bp)** |
| --- | --- | --- | --- | --- |
| 1 | *rs1990429* | Forward | ACGTTGGATGCAGTGAAAAAAATGTGACTC | 104 |
|  |  | Reverse | ACGTTGGATGGACCCTATGTGCTTCTACAG |  |
| 2 | *rs7800545* | Forward | ACGTTGGATGCCCTATCCTTTACACTTCAC | 114 |
|  |  | Reverse | ACGTTGGATGCAACTGTCTGCCAAACAAAG |  |
| 3 | *rs2191736* | Forward | ACGTTGGATGAGTCTGAAGAGGAAATAGGC | 95 |
|  |  | Reverse | ACGTTGGATGTCTGGTCCCACTTACCTTTG |  |
| 4 | *rs17142666* | Forward | ACGTTGGATGGGCAGCAGATAAAAACTTGA | 120 |
|  |  | Reverse | ACGTTGGATGGTTTTACAGATTTTTCAGAC |  |
| 5 | *rs7793864* | Forward | ACGTTGGATGAATTAGGCAGACCTGGGTTC | 103 |
|  |  | Reverse | ACGTTGGATGGGAATCCATGGGAGTAAAGG |  |
| 6 | *rs7810357* | Forward | ACGTTGGATGCACCAATCCATGTTTACCCC | 92 |
|  |  | Reverse | ACGTTGGATGGTTCTCCCTTTTCAGCATTC |  |
| 7 | *rs7793037* | Forward | ACGTTGGATGACCTTTTCATGGAAGACCAG | 118 |
|  |  | Reverse | ACGTTGGATGACCATGTTTCTGTCCTCATC |  |
| 8 | *rs2192373* | Forward | ACGTTGGATGTGGTGTGTCAACGTTAGCAG | 95 |
|  |  | Reverse | ACGTTGGATGGTCTCCTAGAGAACATGGTG |  |
| 9 | *rs802359* | Forward | ACGTTGGATGGAGTTACCTAACACTCTTAC | 113 |
|  |  | Reverse | ACGTTGGATGGCCAACAGTTTTCAAAGCAG |  |
| 10 | *rs802372* | Forward | ACGTTGGATGGGTTCCCACAATCTCAGTTG | 113 |
|  |  | Reverse | ACGTTGGATGGAATAAGCTCCTAGAAGACC |  |
| 11 | *rs1527650* | Forward | ACGTTGGATGTGCTCTGTATATGCAGTGGG | 107 |
|  |  | Reverse | ACGTTGGATGGCTACTTCTCCCTGGATTAC |  |
| 12 | *rs10278347* | Forward | ACGTTGGATGTAAGGCTTCCTTGCTGTTGG | 101 |
|  |  | Reverse | ACGTTGGATGGCAACGGAAATGAAACAACC |  |
| 13 | *rs2402539* | Forward | ACGTTGGATGTTTGCACCTTTGGCTTTCTG | 86 |
|  |  | Reverse | ACGTTGGATGAGGCAGATTCTGATTCAGTC |  |
| 14 | *rs6979618* | Forward | ACGTTGGATGGCCCTTCAACTTGTTTCTCC | 109 |
|  |  | Reverse | ACGTTGGATGCATGAATGGCTTAAGGGAAC |  |
| 15 | *rs7779895* | Forward | ACGTTGGATGCCATTTTTCTAGTTGTCCCG | 114 |
|  |  | Reverse | ACGTTGGATGATTTGGAGGCTACTGGATGG |  |
| 16 | *rs17142875* | Forward | ACGTTGGATGATCGCACAGTGAAAAGCTGG | 110 |
|  |  | Reverse | ACGTTGGATGGGCTTGTCTAGCATGGAATC |  |
| 17 | *rs4727911* | Forward | ACGTTGGATGGTGGTCTAGAGTCTGTTTGC | 115 |
|  |  | Reverse | ACGTTGGATGGCCAAGATTGGAGATTCACC |  |
| 18 | *rs7795646* | Forward | ACGTTGGATGGACATTCTGTGTCTAGAGAG | 95 |
|  |  | Reverse | ACGTTGGATGCTGGTCATTGAGTATACTGC |  |
| 19 | *rs2896298* | Forward | ACGTTGGATGGCAACCACTGGACACTAATG | 100 |
|  |  | Reverse | ACGTTGGATGTCTTCTCCCCCTAACTCTTG |  |
| 20 | *rs1072198* | Forward | ACGTTGGATGGCATTAGTGTCCAGTGGTTG | 99 |
|  |  | Reverse | ACGTTGGATGCTGAAAGTTTATATCTGCTG |  |
| 21 | *rs17142891* | Forward | ACGTTGGATGCAGGCATTACACTGTCAGTC | 85 |
|  |  | Reverse | ACGTTGGATGTGTAGACTAGCTTATAGCTC |  |
| 22 | *rs2189977* | Forward | ACGTTGGATGCGTTATGGTTGATTACATTG | 120 |
|  |  | Reverse | ACGTTGGATGTCTTGAGTGCAAAAGTCCAG |  |
| 23 | *rs11983106* | Forward | ACGTTGGATGCAGGAGCTTTTGTTTTGCTTG | 108 |
|  |  | Reverse | ACGTTGGATGCCAGGCTCCAACATGTTTAG |  |
| 24 | *rs12673992* | Forward | ACGTTGGATGCTATTTCTACTTTGTGACT | 120 |
|  |  | Reverse | ACGTTGGATGCTTTCTATAAGGTTGAAAGG |  |
| 25 | *rs727228* | Forward | ACGTTGGATGTGGGAAGCAAGAATTTCTGG | 120 |
|  |  | Reverse | ACGTTGGATGCCTCTGTCAGAGATGAGATT |  |

**Supplementary Table 6** *KCNB1* tag SNPs in different genetic models associated with ASD risk adjusted by age and sex

| **SNPs ID** | **Model** | **Genotype** | **Control (243)** | **Case (243)** | **Adjusted OR (95 CI)** | ***Adjusted P*** | **AIC** | **BIC** | ***P_FDR_*** |
| --- | --- | --- | --- | --- | --- | --- | --- | --- | --- |
| *rs1051295* | Codominant | A/A | 68 (29.4%) | 59 (25.4%) | 1.00 | 0.630 | 648.6 | 669.3 |  |
|  |  | A/G | 122 (52.8%) | 128 (55.2%) | 1.18 (0.77-1.82) |  |  |  |  |
|  |  | G/G | 41 (17.8%) | 45 (19.4%) | 1.29 (0.74-2.23) |  |  |  |  |
|  | Dominant | A/A | 68 (29.4%) | 59 (25.4%) | 1.00 | 0.360 | 646.7 | 663.3 |  |
|  |  | A/G-G/G | 163 (70.6%) | 173 (74.6%) | 1.21 (0.80-1.82) |  |  |  |  |
|  | Recessive | A/A-A/G | 190 (82.2%) | 187 (80.6%) | 1.00 | 0.560 | 647.2 | 663.8 |  |
|  |  | G/G | 41 (17.8%) | 45 (19.4%) | 1.15 (0.72-1.85) |  |  |  |  |
|  | Over-dominant | A/A-G/G | 109 (47.2%) | 104 (44.8%) | 1.00 | 0.720 | 647.4 | 664.0 |  |
|  |  | A/G | 122 (52.8%) | 128 (55.2%) | 1.07 (0.74-1.55) |  |  |  |  |
|  | **Log-additive** | - | - | - | 1.14 (0.87-1.50) | 0.350 | 646.7 | 663.2 | 0.544 |
| *rs6019774* | Codominant | T/T | 214 (88.8%) | 201 (83.1%) | 1.00 | 0.150 | 673.1 | 694.0 |  |
|  |  | T/C | 25 (10.4%) | 40 (16.5%) | 1.66 (0.97-2.84) |  |  |  |  |
|  |  | C/C | 2 (0.8%) | 1 (0.4%) | 0.55 (0.05-6.15) |  |  |  |  |
|  | Dominant | T/T | 214 (88.8%) | 201 (83.1%) | 1.00 | 0.084 | 672.0 | 688.7 |  |
|  |  | T/C-C/C | 27 (11.2%) | 41 (16.9%) | 1.58 (0.94-2.67) |  |  |  |  |
|  | Recessive | T/T-T/C | 239 (99.2%) | 241 (99.6%) | 1.00 | 0.580 | 674.7 | 691.4 |  |
|  |  | C/C | 2 (0.8%) | 1 (0.4%) | 0.52 (0.05-5.76) |  |  |  |  |
|  | **Over-dominant** | T/T-C/C | 216 (89.6%) | 202 (83.5%) | 1.00 | 0.059 | 671.4 | 688.1 | 0.275 |
|  |  | T/C | 25 (10.4%) | 40 (16.5%) | 1.67 (0.97-2.85) |  |  |  |  |
|  | Log-additive | --- | --- | --- | 1.45 (0.89-2.37) | 0.130 | 672.7 | 689.4 |  |
| *rs2426154* | Codominant | T/T | 86 (36.6%) | 84 (34.9%) | 1.00 | 0.550 | 665.4 | 686.3 |  |
|  |  | C/T | 101 (43.0%) | 115 (47.7%) | 1.14 (0.76-1.71) |  |  |  |  |
|  |  | C/C | 48 (20.4%) | 42 (17.4%) | 0.87 (0.52-1.46) |  |  |  |  |
|  | Dominant | T/T | 86 (36.6%) | 84 (34.9%) | 1.00 | 0.800 | 664.5 | 681.2 |  |
|  |  | C/T-C/C | 149 (63.4%) | 157 (65.2%) | 1.05 (0.72-1.53) |  |  |  |  |
|  | Recessive | T/T-C/T | 187 (79.6%) | 199 (82.6%) | 1.00 | 0.370 | 663.8 | 680.5 |  |
|  |  | C/C | 48 (20.4%) | 42 (17.4%) | 0.81 (0.51-1.29) |  |  |  |  |
|  | **Over-dominant** | T/T-C/C | 134 (57.0%) | 126 (52.3%) | 1.00 | 0.340 | 663.7 | 680.4 | 0.560 |
|  |  | C/T | 101 (43.0%) | 115 (47.7%) | 1.19 (0.83-1.72) |  |  |  |  |
|  | Log-additive | --- | --- | --- | 0.96 (0.75-1.23) | 0.750 | 664.5 | 681.2 |  |
| *rs4810952* | Codominant | T/T | 80 (33.2%) | 87 (36.4%) | 1.00 | 0.360 | 671.3 | 692.2 |  |
|  |  | C/T | 109 (45.2%) | 112 (46.9%) | 0.92 (0.61-1.38) |  |  |  |  |
|  |  | C/C | 52 (21.6%) | 40 (16.7%) | 0.69 (0.42-1.16) |  |  |  |  |
|  | Dominant | T/T | 80 (33.2%) | 87 (36.4%) | 1.00 | 0.390 | 670.6 | 687.3 |  |
|  |  | C/T-C/C | 161 (66.8%) | 152 (63.6%) | 0.85 (0.58-1.24) |  |  |  |  |
|  | **Recessive** | T/T-C/T | 189 (78.4%) | 199 (83.3%) | 1.00 | 0.170 | 669.5 | 686.2 | 0.433 |
|  |  | C/C | 52 (21.6%) | 40 (16.7%) | 0.73 (0.46-1.15) |  |  |  |  |
|  | Over-dominant | T/T-C/C | 132 (54.8%) | 127 (53.1%) | 1.00 | 0.800 | 671.3 | 688.0 |  |
|  |  | C/T | 109 (45.2%) | 112 (46.9%) | 1.05 (0.73-1.50) |  |  |  |  |
|  | Log-additive | --- | --- | --- | 0.84 (0.66-1.09) | 0.190 | 669.6 | 686.3 |  |
| *rs9636516* | Codominant | A/A | 70 (31.4%) | 69 (29.5%) | 1.00 | 0.940 | 639.6 | 660.2 |  |
|  |  | G/A | 103 (46.2%) | 111 (47.4%) | 1.07 (0.70-1.65) |  |  |  |  |
|  |  | G/G | 50 (22.4%) | 54 (23.1%) | 1.06 (0.64-1.77) |  |  |  |  |
|  | **Dominant** | A/A | 70 (31.4%) | 69 (29.5%) | 1.00 | 0.740 | 637.6 | 654.1 | 0.767 |
|  |  | G/A-G/G | 153 (68.6%) | 165 (70.5%) | 1.07 (0.72-1.60) |  |  |  |  |
|  | Recessive | A/A-G/A | 173 (77.6%) | 180 (76.9%) | 1.00 | 0.930 | 637.7 | 654.2 |  |
|  |  | G/G | 50 (22.4%) | 54 (23.1%) | 1.02 (0.66-1.58) |  |  |  |  |
|  | Over-dominant | A/A-G/G | 120 (53.8%) | 123 (52.6%) | 1.00 | 0.810 | 637.7 | 654.2 |  |
|  |  | G/A | 103 (46.2%) | 111 (47.4%) | 1.05 (0.72-1.51) |  |  |  |  |
|  | Log-additive | --- | --- | --- | 1.03 (0.80-1.33) | 0.790 | 637.7 | 654.2 |  |
| *rs756529* | Codominant | A/A | 82 (34.2%) | 91 (37.8%) | 1.00 | 0.510 | 673.1 | 694.0 |  |
|  |  | G/A | 117 (48.8%) | 116 (48.1%) | 0.87 (0.59-1.30) |  |  |  |  |
|  |  | G/G | 41 (17.1%) | 34 (14.1%) | 0.73 (0.42-1.26) |  |  |  |  |
|  | Dominant | A/A | 82 (34.2%) | 91 (37.8%) | 1.00 | 0.340 | 671.6 | 688.3 |  |
|  |  | G/A-G/G | 158 (65.8%) | 150 (62.2%) | 0.83 (0.57-1.22) |  |  |  |  |
|  | Recessive | A/A-G/A | 199 (82.9%) | 207 (85.9%) | 1.00 | 0.350 | 671.6 | 688.3 |  |
|  |  | G/G | 41 (17.1%) | 34 (14.1%) | 0.79 (0.48-1.30) |  |  |  |  |
|  | Over-dominant | A/A-G/G | 123 (51.2%) | 125 (51.9%) | 1.00 | 0.820 | 672.4 | 689.1 |  |
|  |  | G/A | 117 (48.8%) | 116 (48.1%) | 0.96 (0.67-1.38) |  |  |  |  |
|  | **Log-additive** | --- | --- | --- | 0.86 (0.66-1.11) | 0.250 | 671.1 | 687.8 | 0.538 |
| *rs7348799* | **Codominant** | T/T | 214 (88.4%) | 202 (83.1%) | 1.00 | 0.051 | 673.9 | 694.9 | 0.357 |
|  |  | C/T | 26 (10.7%) | 41 (16.9%) | 1.64 (0.97-2.78) |  |  |  |  |
|  |  | C/C | 2 (0.8%) | 0 (0.0%) | 0.00 (0.00-NA) |  |  |  |  |
|  | Dominant | T/T | 214 (88.4%) | 202 (83.1%) | 1.00 | 0.110 | 675.3 | 692.1 |  |
|  |  | C/T-C/C | 28 (11.6%) | 41 (16.9%) | 1.53 (0.91-2.56) |  |  |  |  |
|  | Recessive | T/T-C/T | 240 (99.2%) | 243 (100.0%) | 1.00 | 0.110 | 675.4 | 692.1 |  |
|  |  | C/C | 2 (0.8%) | 0 (0.0%) | 0.00 (0.00-NA) |  |  |  |  |
|  | Over-dominant | T/T-C/C | 216 (89.3%) | 202 (83.1%) | 1.00 | 0.060 | 674.4 | 691.1 |  |
|  |  | C/T | 26 (10.7%) | 41 (16.9%) | 1.65 (0.97-2.80) |  |  |  |  |
|  | Log-additive | --- | --- | --- | 1.39 (0.85-2.27) | 0.190 | 676.2 | 693.0 |  |
| *rs6067087* | Codominant | A/A | 64 (27.2%) | 64 (26.6%) | 1.00 | 0.990 | 667.0 | 687.9 |  |
|  |  | A/G | 111 (47.2%) | 116 (48.1%) | 1.03 (0.66-1.59) |  |  |  |  |
|  |  | G/G | 60 (25.5%) | 61 (25.3%) | 1.00 (0.61-1.65) |  |  |  |  |
|  | **Dominant** | A/A | 64 (27.2%) | 64 (26.6%) | 1.00 | 0.930 | 665.0 | 681.7 | 0.930 |
|  |  | A/G-G/G | 171 (72.8%) | 177 (73.4%) | 1.02 (0.68-1.53) |  |  |  |  |
|  | **Recessive** | A/A-A/G | 175 (74.5%) | 180 (74.7%) | 1.00 | 0.930 | 665.0 | 681.7 | 0.930 |
|  |  | G/G | 60 (25.5%) | 61 (25.3%) | 0.98 (0.65-1.49) |  |  |  |  |
|  | Over-dominant | A/A-G/G | 124 (52.8%) | 125 (51.9%) | 1.00 | 0.880 | 665.0 | 681.7 |  |
|  |  | A/G | 111 (47.2%) | 116 (48.1%) | 1.03 (0.72-1.48) |  |  |  |  |
|  | Log-additive | --- | --- | --- | 1.00 (0.78-1.28) | 1.000 | 665.0 | 681.7 |  |
| *rs6019820* | Codominant | G/G | 85 (35.7%) | 96 (39.8%) | 1.00 | 0.610 | 670.2 | 691.1 |  |
|  |  | G/A | 116 (48.7%) | 112 (46.5%) | 0.85 (0.57-1.25) |  |  |  |  |
|  |  | A/A | 37 (15.6%) | 33 (13.7%) | 0.79 (0.45-1.38) |  |  |  |  |
|  | **Dominant** | G/G | 85 (35.7%) | 96 (39.8%) | 1.00 | 0.330 | 668.3 | 685.0 | 0.578 |
|  |  | G/A-A/A | 153 (64.3%) | 145 (60.2%) | 0.83 (0.57-1.21) |  |  |  |  |
|  | Recessive | G/G-G/A | 201 (84.5%) | 208 (86.3%) | 1.00 | 0.590 | 668.9 | 685.6 |  |
|  |  | A/A | 37 (15.6%) | 33 (13.7%) | 0.87 (0.52-1.45) |  |  |  |  |
|  | Over-dominant | G/G-A/A | 122 (51.3%) | 129 (53.5%) | 1.00 | 0.580 | 668.9 | 685.6 |  |
|  |  | G/A | 116 (48.7%) | 112 (46.5%) | 0.90 (0.63-1.29) |  |  |  |  |
|  | Log-additive | --- | --- | --- | 0.88 (0.68-1.14) | 0.340 | 668.3 | 685.0 |  |
| *rs237459* | Codominant | T/T | 79 (32.9%) | 83 (35.0%) | 1.00 | 0.770 | 668.4 | 689.3 |  |
|  |  | C/T | 111 (46.2%) | 102 (43.0%) | 0.87 (0.58-1.32) |  |  |  |  |
|  |  | C/C | 50 (20.8%) | 52 (21.9%) | 0.99 (0.60-1.63) |  |  |  |  |
|  | Dominant | T/T | 79 (32.9%) | 83 (35.0%) | 1.00 | 0.630 | 666.7 | 683.4 |  |
|  |  | C/T-C/C | 161 (67.1%) | 154 (65.0%) | 0.91 (0.62-1.33) |  |  |  |  |
|  | Recessive | T/T-C/T | 190 (79.2%) | 185 (78.1%) | 1.00 | 0.760 | 666.8 | 683.5 |  |
|  |  | C/C | 50 (20.8%) | 52 (21.9%) | 1.07 (0.69-1.66) |  |  |  |  |
|  | **Over-dominant** | T/T-C/C | 129 (53.8%) | 135 (57.0%) | 1.00 | 0.480 | 666.4 | 683.1 | 0.584 |
|  |  | C/T | 111 (46.2%) | 102 (43.0%) | 0.88 (0.61-1.26) |  |  |  |  |
|  | Log-additive | --- | --- | --- | 0.98 (0.77-1.26) | 0.890 | 666.9 | 683.6 |  |
| *rs237477* | Codominant | T/T | 109 (45.2%) | 119 (49.6%) | 1.00 | 0.200 | 671.0 | 691.9 |  |
|  |  | T/C | 108 (44.8%) | 89 (37.1%) | 0.77 (0.53-1.13) |  |  |  |  |
|  |  | C/C | 24 (10.0%) | 32 (13.3%) | 1.26 (0.70-2.29) |  |  |  |  |
|  | Dominant | T/T | 109 (45.2%) | 119 (49.6%) | 1.00 | 0.410 | 671.6 | 688.3 |  |
|  |  | T/C-C/C | 132 (54.8%) | 121 (50.4%) | 0.86 (0.60-1.23) |  |  |  |  |
|  | Recessive | T/T-T/C | 217 (90.0%) | 208 (86.7%) | 1.00 | 0.220 | 670.7 | 687.4 |  |
|  |  | C/C | 24 (10.0%) | 32 (13.3%) | 1.43 (0.81-2.51) |  |  |  |  |
|  | **Over-dominant** | T/T-C/C | 133 (55.2%) | 151 (62.9%) | 1.00 | 0.100 | 669.6 | 686.3 | 0.350 |
|  |  | T/C | 108 (44.8%) | 89 (37.1%) | 0.74 (0.51-1.06) |  |  |  |  |
|  | Log-additive | --- | --- | --- | 1.00 (0.76-1.30) | 0.980 | 672.3 | 689.0 |  |
| *rs3787318* | Codominant | T/T | 208 (86.7%) | 194 (80.2%) | 1.00 | 0.130 | 671.6 | 692.5 |  |
|  |  | C/T | 30 (12.5%) | 47 (19.4%) | 1.64 (0.99-2.70) |  |  |  |  |
|  |  | C/C | 2 (0.8%) | 1 (0.4%) | 0.54 (0.05-6.09) |  |  |  |  |
|  | Dominant | T/T | 208 (86.7%) | 194 (80.2%) | 1.00 | 0.070 | 670.4 | 687.1 |  |
|  |  | C/T-C/C | 32 (13.3%) | 48 (19.8%) | 1.57 (0.96-2.56) |  |  |  |  |
|  | Recessive | T/T-C/T | 238 (99.2%) | 241 (99.6%) | 1.00 | 0.570 | 673.4 | 690.1 |  |
|  |  | C/C | 2 (0.8%) | 1 (0.4%) | 0.51 (0.05-5.67) |  |  |  |  |
|  | **Over-dominant** | T/T-C/C | 210 (87.5%) | 195 (80.6%) | 1.00 | 0.049 | 669.8 | 686.6 | 0.457 |
|  |  | C/T | 30 (12.5%) | 47 (19.4%) | 1.64 (1.00-2.71) |  |  |  |  |
|  | Log-additive | --- | --- | --- | 1.46 (0.92-2.31) | 0.110 | 671.1 | 687.9 |  |
| *rs742759* | Codominant | G/G | 180 (74.4%) | 170 (70.2%) | 1.00 | 0.550 | 677.5 | 698.5 |  |
|  |  | A/G | 57 (23.6%) | 64 (26.4%) | 1.16 (0.76-1.76) |  |  |  |  |
|  |  | A/A | 5 (2.1%) | 8 (3.3%) | 1.68 (0.54-5.25) |  |  |  |  |
|  | Dominant | G/G | 180 (74.4%) | 170 (70.2%) | 1.00 | 0.370 | 675.9 | 692.7 |  |
|  |  | A/G-A/A | 62 (25.6%) | 72 (29.8%) | 1.20 (0.80-1.79) |  |  |  |  |
|  | Recessive | G/G-A/G | 237 (97.9%) | 234 (96.7%) | 1.00 | 0.400 | 676.0 | 692.7 |  |
|  |  | A/A | 5 (2.1%) | 8 (3.3%) | 1.62 (0.52-5.03) |  |  |  |  |
|  | Over-dominant | G/G-A/A | 185 (76.5%) | 178 (73.5%) | 1.00 | 0.540 | 676.4 | 693.1 |  |
|  |  | A/G | 57 (23.6%) | 64 (26.4%) | 1.14 (0.75-1.72) |  |  |  |  |
|  | **Log-additive** | --- | --- | --- | 1.20 (0.85-1.71) | 0.300 | 675.7 | 692.4 | 0.600 |
| *rs237478* | Codominant | C/C | 76 (31.5%) | 80 (33.1%) | 1.00 | 0.780 | 676.7 | 697.6 |  |
|  |  | T/C | 109 (45.2%) | 102 (42.1%) | 0.90 (0.59-1.36) |  |  |  |  |
|  |  | T/T | 56 (23.2%) | 60 (24.8%) | 1.04 (0.64-1.69) |  |  |  |  |
|  | Dominant | C/C | 76 (31.5%) | 80 (33.1%) | 1.00 | 0.780 | 675.1 | 691.8 |  |
|  |  | T/C-T/T | 165 (68.5%) | 162 (66.9%) | 0.95 (0.65-1.39) |  |  |  |  |
|  | Recessive | C/C-T/C | 185 (76.8%) | 182 (75.2%) | 1.00 | 0.620 | 675.0 | 691.7 |  |
|  |  | T/T | 56 (23.2%) | 60 (24.8%) | 1.11 (0.73-1.69) |  |  |  |  |
|  | **Over-dominant** | C/C-T/T | 132 (54.8%) | 140 (57.9%) | 1.00 | 0.490 | 674.7 | 691.4 | 0.572 |
|  |  | T/C | 109 (45.2%) | 102 (42.1%) | 0.88 (0.61-1.26) |  |  |  |  |
|  | Log-additive | --- | --- | --- | 1.01 (0.80-1.29) | 0.920 | 675.2 | 691.9 |  |
| *rs13044742* | Codominant | A/A | 185 (77.7%) | 183 (79.6%) | 1.00 | 0.700 | 655.2 | 676.0 |  |
|  |  | A/T | 49 (20.6%) | 42 (18.3%) | 0.84 (0.53-1.34) |  |  |  |  |
|  |  | T/T | 4 (1.7%) | 5 (2.2%) | 1.30 (0.34-4.93) |  |  |  |  |
|  | Dominant | A/A | 185 (77.7%) | 183 (79.6%) | 1.00 | 0.560 | 653.6 | 670.2 |  |
|  |  | A/T-T/T | 53 (22.3%) | 47 (20.4%) | 0.88 (0.56-1.37) |  |  |  |  |
|  | Recessive | A/A-A/T | 234 (98.3%) | 225 (97.8%) | 1.00 | 0.670 | 653.8 | 670.4 |  |
|  |  | T/T | 4 (1.7%) | 5 (2.2%) | 1.34 (0.35-5.08) |  |  |  |  |
|  | **Over-dominant** | A/A-T/T | 189 (79.4%) | 188 (81.7%) | 1.00 | 0.450 | 653.4 | 670.0 | 0.573 |
|  |  | A/T | 49 (20.6%) | 42 (18.3%) | 0.84 (0.53-1.33) |  |  |  |  |
|  | Log-additive | --- | --- | --- | 0.93 (0.63-1.37) | 0.700 | 653.8 | 670.4 |  |
| *rs572845* | **Codominant** | G/G | 182 (74.9%) | 191 (78.9%) | 1.00 | 0.036 | 673.0 | 693.9 | 0.504 |
|  |  | G/A | 60 (24.7%) | 45 (18.6%) | 0.70 (0.45-1.08) |  |  |  |  |
|  |  | A/A | 1 (0.4%) | 6 (2.5%) | 5.69 (0.67-47.93) |  |  |  |  |
|  | Dominant | G/G | 182 (74.9%) | 191 (78.9%) | 1.00 | 0.240 | 676.3 | 693.0 |  |
|  |  | G/A-A/A | 61 (25.1%) | 51 (21.1%) | 0.78 (0.51-1.19) |  |  |  |  |
|  | Recessive | G/G-G/A | 242 (99.6%) | 236 (97.5%) | 1.00 | 0.045 | 673.6 | 690.3 |  |
|  |  | A/A | 1 (0.4%) | 6 (2.5%) | 6.16 (0.73-51.72) |  |  |  |  |
|  | Over-dominant | G/G-A/A | 183 (75.3%) | 197 (81.4%) | 1.00 | 0.081 | 674.6 | 691.3 |  |
|  |  | G/A | 60 (24.7%) | 45 (18.6%) | 0.68 (0.44-1.05) |  |  |  |  |
|  | Log-additive | --- | --- | --- | 0.89 (0.61-1.32) | 0.570 | 677.3 | 694.0 |  |
| *rs610412* | Codominant | A/A | 168 (69.7%) | 167 (69.0%) | 1.00 | 0.730 | 676.5 | 697.4 |  |
|  |  | C/A | 68 (28.2%) | 67 (27.7%) | 0.96 (0.65-1.44) |  |  |  |  |
|  |  | C/C | 5 (2.1%) | 8 (3.3%) | 1.55 (0.49-4.85) |  |  |  |  |
|  | Dominant | A/A | 168 (69.7%) | 167 (69.0%) | 1.00 | 0.980 | 675.2 | 691.9 |  |
|  |  | C/A-C/C | 73 (30.3%) | 75 (31.0%) | 1.00 (0.68-1.48) |  |  |  |  |
|  | **Recessive** | A/A-C/A | 236 (97.9%) | 234 (96.7%) | 1.00 | 0.430 | 674.6 | 691.3 | 0.573 |
|  |  | C/C | 5 (2.1%) | 8 (3.3%) | 1.56 (0.50-4.87) |  |  |  |  |
|  | Over-dominant | A/A-C/C | 173 (71.8%) | 175 (72.3%) | 1.00 | 0.800 | 675.1 | 691.8 |  |
|  |  | C/A | 68 (28.2%) | 67 (27.7%) | 0.95 (0.64-1.42) |  |  |  |  |
|  | Log-additive | --- | --- | --- | 1.05 (0.74-1.47) | 0.790 | 675.1 | 691.8 |  |
| *rs6019855* | Codominant | T/T | 124 (52.1%) | 126 (52.1%) | 1.00 | 0.220 | 669.6 | 690.5 |  |
|  |  | G/T | 100 (42.0%) | 92 (38.0%) | 0.89 (0.61-1.29) |  |  |  |  |
|  |  | G/G | 14 (5.9%) | 24 (9.9%) | 1.67 (0.82-3.40) |  |  |  |  |
|  | Dominant | T/T | 124 (52.1%) | 126 (52.1%) | 1.00 | 0.910 | 670.6 | 687.3 |  |
|  |  | G/T-G/G | 114 (47.9%) | 116 (47.9%) | 0.98 (0.68-1.41) |  |  |  |  |
|  | **Recessive** | T/T-G/T | 224 (94.1%) | 218 (90.1%) | 1.00 | 0.100 | 668.0 | 684.7 | 0.311 |
|  |  | G/G | 14 (5.9%) | 24 (9.9%) | 1.76 (0.88-3.51) |  |  |  |  |
|  | Over-dominant | T/T-G/G | 138 (58.0%) | 150 (62.0%) | 1.00 | 0.320 | 669.6 | 686.3 |  |
|  |  | G/T | 100 (42.0%) | 92 (38.0%) | 0.83 (0.57-1.20) |  |  |  |  |
|  | Log-additive | --- | --- | --- | 1.09 (0.82-1.45) | 0.550 | 670.3 | 687.0 |  |
| *rs10485612* | Codominant | A/A | 205 (86.5%) | 196 (81.3%) | 1.00 | 0.340 | 667.9 | 688.7 |  |
|  |  | G/A | 30 (12.7%) | 42 (17.4%) | 1.43 (0.86-2.38) |  |  |  |  |
|  |  | G/G | 2 (0.8%) | 3 (1.2%) | 1.67 (0.27-10.22) |  |  |  |  |
|  | **Dominant** | A/A | 205 (86.5%) | 196 (81.3%) | 1.00 | 0.140 | 665.9 | 682.6 | 0.392 |
|  |  | G/A-G/G | 32 (13.5%) | 45 (18.7%) | 1.44 (0.88-2.37) |  |  |  |  |
|  | Recessive | A/A-G/A | 235 (99.2%) | 238 (98.8%) | 1.00 | 0.610 | 667.8 | 684.5 |  |
|  |  | G/G | 2 (0.8%) | 3 (1.2%) | 1.59 (0.26-9.72) |  |  |  |  |
|  | Over-dominant | A/A-G/G | 207 (87.3%) | 199 (82.6%) | 1.00 | 0.170 | 666.2 | 682.9 |  |
|  |  | G/A | 30 (12.7%) | 42 (17.4%) | 1.42 (0.85-2.37) |  |  |  |  |
|  | **Log-additive** | --- | --- | --- | 1.40 (0.89-2.20) | 0.140 | 665.9 | 682.6 |  |
| *rs802950* | Codominant | C/C | 162 (67.8%) | 170 (70.2%) | 1.00 | 0.620 | 673.1 | 694.0 |  |
|  |  | C/A | 70 (29.3%) | 68 (28.1%) | 0.91 (0.61-1.35) |  |  |  |  |
|  |  | A/A | 7 (2.9%) | 4 (1.6%) | 0.57 (0.16-1.99) |  |  |  |  |
|  | Dominant | C/C | 162 (67.8%) | 170 (70.2%) | 1.00 | 0.510 | 671.7 | 688.4 |  |
|  |  | C/A-A/A | 77 (32.2%) | 72 (29.8%) | 0.88 (0.59-1.29) |  |  |  |  |
|  | **Recessive** | C/C-C/A | 232 (97.1%) | 238 (98.3%) | 1.00 | 0.390 | 671.4 | 688.1 | 0.575 |
|  |  | A/A | 7 (2.9%) | 4 (1.6%) | 0.58 (0.17-2.03) |  |  |  |  |
|  | Over-dominant | C/C-A/A | 169 (70.7%) | 174 (71.9%) | 1.00 | 0.690 | 671.9 | 688.6 |  |
|  |  | C/A | 70 (29.3%) | 68 (28.1%) | 0.92 (0.62-1.37) |  |  |  |  |
|  | Log-additive | --- | --- | --- | 0.86 (0.61-1.22) | 0.400 | 671.4 | 688.1 |  |
| *rs653070* | Codominant | C/C | 102 (43.0%) | 104 (43.0%) | 1.00 | 0.920 | 670.9 | 691.7 |  |
|  |  | C/T | 111 (46.8%) | 111 (45.9%) | 0.95 (0.65-1.39) |  |  |  |  |
|  |  | T/T | 24 (10.1%) | 27 (11.2%) | 1.07 (0.58-1.99) |  |  |  |  |
|  | Dominant | C/C | 102 (43.0%) | 104 (43.0%) | 1.00 | 0.870 | 669.0 | 685.7 |  |
|  |  | C/T-T/T | 135 (57.0%) | 138 (57.0%) | 0.97 (0.67-1.40) |  |  |  |  |
|  | Recessive | C/C-C/T | 213 (89.9%) | 215 (88.8%) | 1.00 | 0.750 | 669.0 | 685.6 |  |
|  |  | T/T | 24 (10.1%) | 27 (11.2%) | 1.10 (0.61-1.97) |  |  |  |  |
|  | **Over-dominant** | C/C-T/T | 126 (53.2%) | 131 (54.1%) | 1.00 | 0.720 | 668.9 | 685.6 | 0.775 |
|  |  | C/T | 111 (46.8%) | 111 (45.9%) | 0.94 (0.65-1.35) |  |  |  |  |
|  | Log-additive | --- | --- | --- | 1.00 (0.76-1.32) | 0.980 | 669.1 | 685.7 |  |
| *rs4809745* | Codominant | G/G | 194 (80.8%) | 178 (74.8%) | 1.00 | 0.240 | 667.8 | 688.7 |  |
|  |  | G/A | 42 (17.5%) | 57 (23.9%) | 1.45 (0.93-2.28) |  |  |  |  |
|  |  | A/A | 4 (1.7%) | 3 (1.3%) | 0.77 (0.17-3.51) |  |  |  |  |
|  | Dominant | G/G | 194 (80.8%) | 178 (74.8%) | 1.00 | 0.140 | 666.5 | 683.2 |  |
|  |  | G/A-A/A | 46 (19.2%) | 60 (25.2%) | 1.39 (0.90-2.16) |  |  |  |  |
|  | Recessive | G/G-G/A | 236 (98.3%) | 235 (98.7%) | 1.00 | 0.660 | 668.5 | 685.2 |  |
|  |  | A/A | 4 (1.7%) | 3 (1.3%) | 0.71 (0.16-3.22) |  |  |  |  |
|  | **Over-dominant** | G/G-A/A | 198 (82.5%) | 181 (76.0%) | 1.00 | 0.096 | 665.9 | 682.6 | 0.384 |
|  |  | G/A | 42 (17.5%) | 57 (23.9%) | 1.46 (0.93-2.28) |  |  |  |  |
|  | Log-additive | --- | --- | --- | 1.28 (0.86-1.91) | 0.220 | 667.2 | 683.9 |  |
| *rs552068* | Codominant | A/A | 77 (31.9%) | 69 (28.6%) | 1.00 | 0.840 | 675.1 | 696.0 |  |
|  |  | C/A | 117 (48.5%) | 121 (50.2%) | 1.11 (0.73-1.68) |  |  |  |  |
|  |  | C/C | 47 (19.5%) | 51 (21.2%) | 1.16 (0.69-1.94) |  |  |  |  |
|  | Dominant | A/A | 77 (31.9%) | 69 (28.6%) | 1.00 | 0.570 | 673.2 | 689.9 |  |
|  |  | C/A-C/C | 164 (68.0%) | 172 (71.4%) | 1.12 (0.76-1.66) |  |  |  |  |
|  | Recessive | A/A-C/A | 194 (80.5%) | 190 (78.8%) | 1.00 | 0.710 | 673.3 | 690.1 |  |
|  |  | C/C | 47 (19.5%) | 51 (21.2%) | 1.09 (0.70-1.70) |  |  |  |  |
|  | Over-dominant | A/A-C/C | 124 (51.5%) | 120 (49.8%) | 1.00 | 0.830 | 673.4 | 690.1 |  |
|  |  | C/A | 117 (48.5%) | 121 (50.2%) | 1.04 (0.73-1.49) |  |  |  |  |
|  | **Log-additive** | --- | --- | --- | 1.08 (0.83-1.39) | 0.560 | 673.1 | 689.9 | 0.672 |
| *rs6125656* | Codominant | G/G | 207 (86.6%) | 202 (83.5%) | 1.00 | 0.350 | 672.0 | 692.9 |  |
|  |  | A/G | 32 (13.4%) | 39 (16.1%) | 1.24 (0.75-2.06) |  |  |  |  |
|  |  | A/A | 0 (0.0%) | 1 (0.4%) | NA (0.00-NA) |  |  |  |  |
|  | Dominant | G/G | 207 (86.6%) | 202 (83.5%) | 1.00 | 0.350 | 671.2 | 687.9 |  |
|  |  | A/G-A/A | 32 (13.4%) | 40 (16.5%) | 1.27 (0.77-2.11) |  |  |  |  |
|  | **Recessive** | G/G-A/G | 239 (100.0%) | 241 (99.6%) | 1.00 | 0.240 | 670.7 | 687.4 | 0.560 |
|  |  | A/A | 0 (0.0%) | 1 (0.4%) | NA (0.00-NA) |  |  |  |  |
|  | Over-dominant | G/G-A/A | 207 (86.6%) | 203 (83.9%) | 1.00 | 0.420 | 671.4 | 688.1 |  |
|  |  | A/G | 32 (13.4%) | 39 (16.1%) | 1.23 (0.74-2.05) |  |  |  |  |
|  | Log-additive | --- | --- | --- | 1.30 (0.79-2.14) | 0.300 | 671.0 | 687.7 |  |
| *rs477135* | Codominant | T/T | 167 (70.2%) | 147 (61.5%) | 1.00 | 0.031 | 662.0 | 682.8 |  |
|  |  | A/T | 57 (23.9%) | 83 (34.7%) | **1.63 (1.09-2.44)** |  |  |  |  |
|  |  | A/A | 14 (5.9%) | 9 (3.8%) | 0.70 (0.29-1.68) |  |  |  |  |
|  | Dominant | T/T | 167 (70.2%) | 147 (61.5%) | 1.00 | 0.059 | 663.4 | 680.0 |  |
|  |  | A/T-A/A | 71 (29.8%) | 92 (38.5%) | 1.44 (0.99-2.12) |  |  |  |  |
|  | Recessive | T/T-A/T | 224 (94.1%) | 230 (96.2%) | 1.00 | 0.240 | 665.6 | 682.2 |  |
|  |  | A/A | 14 (5.9%) | 9 (3.8%) | 0.60 (0.26-1.43) |  |  |  |  |
|  | **Over-dominant** | T/T-A/A | 181 (76.0%) | 156 (65.3%) | 1.00 | 0.012 | 660.6 | 677.3 | 0.336 |
|  |  | A/T | 57 (23.9%) | 83 (34.7%) | **1.67 (1.12-2.49)** |  |  |  |  |
|  | Log-additive | --- | --- | --- | 1.20 (0.87-1.64) | 0.260 | 665.7 | 682.3 |  |
| *rs566604* | Codominant | A/A | 170 (70.5%) | 150 (62.0%) | 1.00 | 0.150 | 673.4 | 694.3 |  |
|  |  | G/A | 63 (26.1%) | 84 (34.7%) | 1.48 (1.00-2.20) |  |  |  |  |
|  |  | G/G | 8 (3.3%) | 8 (3.3%) | 1.11 (0.41-3.03) |  |  |  |  |
|  | Dominant | A/A | 170 (70.5%) | 150 (62.0%) | 1.00 | 0.061 | 671.7 | 688.4 |  |
|  |  | G/A-G/G | 71 (29.5%) | 92 (38.0%) | 1.44 (0.98-2.11) |  |  |  |  |
|  | Recessive | A/A-G/A | 233 (96.7%) | 234 (96.7%) | 1.00 | 0.970 | 675.2 | 691.9 |  |
|  |  | G/G | 8 (3.3%) | 8 (3.3%) | 0.98 (0.36-2.66) |  |  |  |  |
|  | **Over-dominant** | A/A-G/G | 178 (73.9%) | 158 (65.3%) | 1.00 | 0.052 | 671.4 | 688.1 | 0.291 |
|  |  | G/A | 63 (26.1%) | 84 (34.7%) | 1.47 (0.99-2.18) |  |  |  |  |
|  | Log-additive | --- | --- | --- | 1.31 (0.94-1.82) | 0.110 | 672.6 | 689.3 |  |
| *rs7269864* | Codominant | T/T | 192 (80.0%) | 184 (76.0%) | 1.00 | 0.590 | 674.8 | 695.6 |  |
|  |  | C/T | 43 (17.9%) | 53 (21.9%) | 1.27 (0.81-1.99) |  |  |  |  |
|  |  | C/C | 5 (2.1%) | 5 (2.1%) | 0.99 (0.28-3.48) |  |  |  |  |
|  | Dominant | T/T | 192 (80.0%) | 184 (76.0%) | 1.00 | 0.340 | 672.9 | 689.6 |  |
|  |  | C/T-C/C | 48 (20.0%) | 58 (24.0%) | 1.24 (0.80-1.91) |  |  |  |  |
|  | Recessive | T/T-C/T | 235 (97.9%) | 237 (97.9%) | 1.00 | 0.920 | 673.8 | 690.5 |  |
|  |  | C/C | 5 (2.1%) | 5 (2.1%) | 0.94 (0.27-3.30) |  |  |  |  |
|  | **Over-dominant** | T/T-C/C | 197 (82.1%) | 189 (78.1%) | 1.00 | 0.300 | 672.8 | 689.5 | 0.560 |
|  |  | C/T | 43 (17.9%) | 53 (21.9%) | 1.27 (0.81-1.99) |  |  |  |  |
|  | Log-additive | --- | --- | --- | 1.17 (0.80-1.71) | 0.420 | 673.2 | 689.9 |  |
| *rs553213* | Codominant | A/A | 101 (42.6%) | 93 (38.9%) | 1.00 | 0.690 | 666.6 | 687.4 |  |
|  |  | G/A | 103 (43.5%) | 106 (44.4%) | 1.09 (0.74-1.62) |  |  |  |  |
|  |  | G/G | 33 (13.9%) | 40 (16.7%) | 1.27 (0.74-2.18) |  |  |  |  |
|  | Dominant | A/A | 101 (42.6%) | 93 (38.9%) | 1.00 | 0.500 | 664.9 | 681.6 |  |
|  |  | G/A-G/G | 136 (57.4%) | 146 (61.1%) | 1.13 (0.79-1.64) |  |  |  |  |
|  | Recessive | A/A-G/A | 204 (86.1%) | 199 (83.3%) | 1.00 | 0.460 | 664.8 | 681.5 |  |
|  |  | G/G | 33 (13.9%) | 40 (16.7%) | 1.21 (0.73-2.00) |  |  |  |  |
|  | Over-dominant | A/A-G/G | 134 (56.5%) | 133 (55.6%) | 1.00 | 0.900 | 665.3 | 682.0 |  |
|  |  | G/A | 103 (43.5%) | 106 (44.4%) | 1.02 (0.71-1.47) |  |  |  |  |
|  | **Log-additive** | --- | --- | --- | 1.12 (0.86-1.45) | 0.400 | 664.6 | 681.3 | 0.560 |

OR, Odds ratio; CI, confidence interval; AIC, Akaike’ information criterion; BIC, Bayesian information criterion; *p_FDR_*: FDR corrected *p* value; **p* < 0.05; NA is not applicable.

**Supplementary Table 7** *KCND2* tag SNPs in different genetic models associated with ASD risk adjusted by age and sex

| **SNPs ID** | **Model** | **Genotype** | **Control (243)** | **Case (243)** | **OR (95 CI)** | ***P*** | **AIC** | **BIC** | ***P_FDR_*** |
| --- | --- | --- | --- | --- | --- | --- | --- | --- | --- |
| *rs1990429* | Codominant | G/G | 180 (75.3%) | 212 (87.6%) | 1.00 | <0.0001 | 652.6 | 673.4 |  |
|  |  | G/A | 59 (24.7%) | 26 (10.7%) | **0.37 (0.22-0.61)** |  |  |  |  |
|  |  | A/A | 0 (0.0%) | 4 (1.6%) | NA (0.00-NA) |  |  |  |  |
|  | Dominant | G/G | 180 (75.3%) | 212 (87.6%) | 1.00 | <0.0001 | 659.3 | 676.0 |  |
|  |  | G/A-A/A | 59 (24.7%) | 30 (12.4%) | **0.42 (0.26-0.68)** |  |  |  |  |
|  | Recessive | G/G-G/A | 239 (100.0%) | 238 (98.3%) | 1.00 | 0.022 | 666.9 | 683.6 |  |
|  |  | A/A | 0 (0.0%) | 4 (1.6%) | NA (0.00-NA) |  |  |  |  |
|  | **Over-dominant** | G/G-A/A | 180 (75.3%) | 216 (89.3%) | 1.00 | <0.0001 | 655.1 | 671.8 | **<0.001*** |
|  |  | G/A | 59 (24.7%) | 26 (10.7%) | **0.36 (0.22-0.60)** |  |  |  |  |
|  | Log-additive | --- | --- | --- | **0.52 (0.33-0.81)** | 0.0035 | 663.6 | 680.3 |  |
| *rs7800545* | Codominant | A/A | 194 (80.5%) | 214 (89.2%) | 1.00 | 0.024 | 666.9 | 687.8 |  |
|  |  | G/A | 44 (18.3%) | 24 (10.0%) | **0.49 (0.28-0.83)** |  |  |  |  |
|  |  | G/G | 3 (1.2%) | 2 (0.8%) | 0.59 (0.10-3.58) |  |  |  |  |
|  | **Dominant** | A/A | 194 (80.5%) | 214 (89.2%) | 1.00 | 0.0064 | 665.0 | 681.7 | **0.045*** |
|  |  | G/A-G/G | 47 (19.5%) | 26 (10.8%) | **0.49 (0.29-0.83)** |  |  |  |  |
|  | Recessive | A/A-G/A | 238 (98.8%) | 238 (99.2%) | 1.00 | 0.640 | 672.2 | 688.9 |  |
|  |  | G/G | 3 (1.2%) | 2 (0.8%) | 0.65 (0.11-3.96) |  |  |  |  |
|  | Over-dominant | A/A-G/G | 197 (81.7%) | 216 (90.0%) | 1.00 | 0.0076 | 665.3 | 682.0 |  |
|  |  | G/A | 44 (18.3%) | 24 (10.0%) | **0.49 (0.29-0.84)** |  |  |  |  |
|  | Log-additive | --- | --- | --- | **0.54 (0.33-0.87)** | 0.0093 | 665.6 | 682.3 |  |
| *rs17142666* | Codominant | G/G | 104 (44.3%) | 106 (44.4%) | 1.00 | 0.990 | 664.4 | 685.2 |  |
|  |  | A/G | 106 (45.1%) | 108 (45.2%) | 0.99 (0.67-1.44) |  |  |  |  |
|  |  | A/A | 25 (10.6%) | 25 (10.5%) | 0.97 (0.52-1.80) |  |  |  |  |
|  | Dominant | G/G | 104 (44.3%) | 106 (44.4%) | 1.00 | 0.930 | 662.4 | 679.0 |  |
|  |  | A/G-A/A | 131 (55.7%) | 133 (55.6%) | 0.98 (0.68-1.41) |  |  |  |  |
|  | Recessive | G/G-A/G | 210 (89.4%) | 214 (89.5%) | 1.00 | 0.940 | 662.4 | 679.0 |  |
|  |  | A/A | 25 (10.6%) | 25 (10.5%) | 0.98 (0.54-1.76) |  |  |  |  |
|  | Over-dominant | G/G-A/A | 129 (54.9%) | 131 (54.8%) | 1.00 | 0.960 | 662.4 | 679.0 |  |
|  |  | A/G | 106 (45.1%) | 108 (45.2%) | 0.99 (0.69-1.43) |  |  |  |  |
|  | **Log-additive** | --- | --- | --- | 0.99 (0.75-1.30) | 0.920 | 662.4 | 679.0 | 0.966 |
| *rs7793864* | Codominant | T/T | 213 (88.4%) | 235 (97.5%) | 1.00 | <0.0001 | 654.7 | 675.5 |  |
|  |  | A/T | 28 (11.6%) | 5 (2.1%) | **0.16 (0.06-0.41)** |  |  |  |  |
|  |  | A/A | 0 (0.0%) | 1 (0.4%) | NA (0.00-NA) |  |  |  |  |
|  | Dominant | T/T | 213 (88.4%) | 235 (97.5%) | 1.00 | <0.0001 | 656.3 | 673.0 |  |
|  |  | A/T-A/A | 28 (11.6%) | 6 (2.5%) | **0.19 (0.08-0.46)** |  |  |  |  |
|  | Recessive | T/T-A/T | 241 (100.0%) | 240 (99.6%) | 1.00 | 0.240 | 672.2 | 688.9 |  |
|  |  | A/A | 0 (0.0%) | 1 (0.4%) | NA (0.00-NA) |  |  |  |  |
|  | **Over-dominant** | T/T-A/A | 213 (88.4%) | 236 (97.9%) | 1.00 | <0.0001 | 653.9 | 670.6 | **<0.001*** |
|  |  | A/T | 28 (11.6%) | 5 (2.1%) | **0.15 (0.06-0.41)** |  |  |  |  |
|  | Log-additive | --- | --- | --- | **0.23 (0.10-0.54)** | <0.0001 | 659.1 | 675.8 |  |
| *rs7810357* | Codominant | G/G | 205 (86.1%) | 222 (92.5%) | 1.00 | 0.040 | 663.0 | 683.9 |  |
|  |  | G/A | 32 (13.4%) | 18 (7.5%) | **0.51 (0.28-0.94)** |  |  |  |  |
|  |  | A/A | 1 (0.4%) | 0 (0.0%) | 0.00 (0.00-NA) |  |  |  |  |
|  | Dominant | G/G | 205 (86.1%) | 222 (92.5%) | 1.00 | 0.019 | 662.0 | 678.6 |  |
|  |  | G/A-A/A | 33 (13.9%) | 18 (7.5%) | **0.49 (0.27-0.90)** |  |  |  |  |
|  | Recessive | G/G-G/A | 237 (99.6%) | 240 (100.0%) | 1.00 | 0.220 | 665.9 | 682.6 |  |
|  |  | A/A | 1 (0.4%) | 0 (0%) | 0.00 (0.00-NA) |  |  |  |  |
|  | Over-dominant | G/G-A/A | 206 (86.5%) | 222 (92.5%) | 1.00 | 0.028 | 662.6 | 679.3 |  |
|  |  | G/A | 32 (13.4%) | 18 (7.5%) | **0.51 (0.28-0.94)** |  |  |  |  |
|  | **Log-additive** | --- | --- | --- | **0.49 (0.27-0.88)** | 0.015 | 661.5 | 678.2 | 0.079 |
| *rs7793037* | Codominant | A/A | 65 (27.1%) | 76 (31.9%) | 1.00 | 0.240 | 667.5 | 688.3 |  |
|  |  | G/A | 117 (48.8%) | 117 (49.2%) | 0.84 (0.55-1.28) |  |  |  |  |
|  |  | G/G | 58 (24.2%) | 45 (18.9%) | 0.65 (0.39-1.08) |  |  |  |  |
|  | Dominant | A/A | 65 (27.1%) | 76 (31.9%) | 1.00 | 0.21 | 666.7 | 683.4 |  |
|  |  | G/A-G/G | 175 (72.9%) | 162 (68.1%) | 0.78 (0.52-1.16) |  |  |  |  |
|  | Recessive | A/A-G/A | 182 (75.8%) | 193 (81.1%) | 1.00 | 0.140 | 666.1 | 682.8 |  |
|  |  | G/G | 58 (24.2%) | 45 (18.9%) | 0.72 (0.46-1.12) |  |  |  |  |
|  | Over-dominant | A/A-G/G | 123 (51.2%) | 121 (50.8%) | 1.00 | 0.940 | 668.3 | 685.0 |  |
|  |  | G/A | 117 (48.8%) | 117 (49.2%) | 1.01 (0.71-1.45) |  |  |  |  |
|  | **Log-additive** | --- | --- | --- | 0.81 (0.62-1.04) | 0.098 | 665.5 | 682.2 | 0.206 |
| *rs2192373* | --- | C/C | 209 (87.5%) | 217 (89.7%) | 1.00 | 0.380 | 671.3 | 688.0 |  |
|  |  | T/C | 30 (12.6%) | 25 (10.3%) | 0.78 (0.44-1.37) |  |  |  |  |
| *rs802372* | Codominant | A/A | 72 (30.2%) | 60 (24.9%) | 1.00 | 0.380 | 669.4 | 690.3 |  |
|  |  | G/A | 108 (45.4%) | 122 (50.6%) | 1.35 (0.88-2.08) |  |  |  |  |
|  |  | G/G | 58 (24.4%) | 59 (24.5%) | 1.22 (0.74-2.01) |  |  |  |  |
|  | **Dominant** | A/A | 72 (30.2%) | 60 (24.9%) | 1.00 | 0.190 | 667.7 | 684.3 | 0.307 |
|  |  | G/A-G/G | 166 (69.8%) | 181 (75.1%) | 1.31 (0.87-1.96) |  |  |  |  |
|  | Recessive | A/A-G/A | 180 (75.6%) | 182 (75.5%) | 1.00 | 0.980 | 669.4 | 686.0 |  |
|  |  | G/G | 58 (24.4%) | 59 (24.5%) | 1.01 (0.66-1.53) |  |  |  |  |
|  | Over-dominant | A/A-G/G | 130 (54.6%) | 119 (49.4%) | 1.00 | 0.250 | 668.1 | 684.7 |  |
|  |  | G/A | 108 (45.4%) | 122 (50.6%) | 1.23 (0.86-1.77) |  |  |  |  |
|  | Log-additive | --- | --- | --- | 1.11 (0.87-1.43) | 0.410 | 668.7 | 685.4 |  |
| *rs1527650* | Codominant | T/T | 221 (92.9%) | 225 (93.4%) | 1.00 | 0.460 | 669.3 | 690.2 |  |
|  |  | G/T | 16 (6.7%) | 16 (6.6%) | 0.95 (0.46-1.96) |  |  |  |  |
|  |  | G/G | 1 (0.4%) | 0 (0.0%) | 0.00 (0.00-NA) |  |  |  |  |
|  | Dominant | T/T | 221 (92.9%) | 225 (93.4%) | 1.00 | 0.760 | 668.7 | 685.4 |  |
|  |  | G/T-G/G | 17 (7.1%) | 16 (6.6%) | 0.89 (0.44-1.82) |  |  |  |  |
|  | **Recessive** | T/T-G/T | 237 (99.6%) | 241 (100.0%) | 1.00 | 0.220 | 667.3 | 684.0 | 0.308 |
|  |  | G/G | 1 (0.4%) | 0 (0.0%) | 0.00 (0.00-NA) |  |  |  |  |
|  | Over-dominant | T/T-G/G | 222 (93.3%) | 225 (93.4%) | 1.00 | 0.910 | 668.8 | 685.5 |  |
|  |  | G/T | 16 (6.7%) | 16 (6.6%) | 0.96 (0.47-1.97) |  |  |  |  |
|  | Log-additive | --- | --- | --- | 0.85 (0.43-1.67) | 0.630 | 668.6 | 685.3 |  |
| *rs10278347* | Codominant | C/C | 71 (29.8%) | 55 (23.0%) | 1.00 | 0.220 | 665.4 | 686.2 |  |
|  |  | C/A | 110 (46.2%) | 116 (48.5%) | 1.36 (0.88-2.11) |  |  |  |  |
|  |  | A/A | 57 (23.9%) | 68 (28.4%) | 1.53 (0.93-2.51) |  |  |  |  |
|  | **Dominant** | C/C | 71 (29.8%) | 55 (23.0%) | 1.00 | 0.096 | 663.6 | 680.3 | 0.224 |
|  |  | C/A-A/A | 167 (70.2%) | 184 (77.0%) | 1.42 (0.94-2.14) |  |  |  |  |
|  | Recessive | C/C-C/A | 181 (76%) | 171 (71.5%) | 1.00 | 0.280 | 665.3 | 681.9 |  |
|  |  | A/A | 57 (23.9%) | 68 (28.4%) | 1.25 (0.83-1.89) |  |  |  |  |
|  | Over-dominant | C/C-A/A | 128 (53.8%) | 123 (51.5%) | 1.00 | 0.600 | 666.1 | 682.8 |  |
|  |  | C/A | 110 (46.2%) | 116 (48.5%) | 1.10 (0.77-1.58) |  |  |  |  |
|  | Log-additive | --- | --- | --- | 1.24 (0.96-1.59) | 0.096 | 663.6 | 680.3 |  |
| *rs2402539* | Codominant | T/T | 66 (27.9%) | 54 (22.9%) | 1.00 | 0.260 | 660.6 | 681.4 |  |
|  |  | C/T | 110 (46.4%) | 127 (53.8%) | 1.41 (0.91-2.20) |  |  |  |  |
|  |  | C/C | 61 (25.7%) | 55 (23.3%) | 1.11 (0.66-1.85) |  |  |  |  |
|  | Dominant | T/T | 66 (27.9%) | 54 (22.9%) | 1.00 | 0.210 | 659.7 | 676.4 |  |
|  |  | C/T-C/C | 171 (72.2%) | 182 (77.1%) | 1.30 (0.86-1.98) |  |  |  |  |
|  | Recessive | T/T-C/T | 176 (74.3%) | 181 (76.7%) | 1.00 | 0.560 | 660.9 | 677.6 |  |
|  |  | C/C | 61 (25.7%) | 55 (23.3%) | 0.88 (0.58-1.34) |  |  |  |  |
|  | **Over-dominant** | T/T-C/C | 127 (53.6%) | 109 (46.2%) | 1.00 | 0.110 | 658.7 | 675.4 | 0.21 |
|  |  | C/T | 110 (46.4%) | 127 (53.8%) | 1.34 (0.93-1.93) |  |  |  |  |
|  | Log-additive | --- | --- | --- | 1.05 (0.82-1.36) | 0.680 | 661.1 | 677.8 |  |
| *rs6979618* | Codominant | A/A | 68 (28.3%) | 82 (34.3%) | 1.00 | 0.058 | 665.8 | 686.7 |  |
|  |  | A/G | 112 (46.7%) | 118 (49.4%) | 0.86 (0.57-1.30) |  |  |  |  |
|  |  | G/G | 60 (25.0%) | 39 (16.3%) | **0.54 (0.32-0.91)** |  |  |  |  |
|  | Dominant | A/A | 68 (28.3%) | 82 (34.3%) | 1.00 | 0.150 | 667.4 | 684.1 |  |
|  |  | A/G-G/G | 172 (71.7%) | 157 (65.7%) | 0.75 (0.51-1.11) |  |  |  |  |
|  | **Recessive** | A/A-A/G | 180 (75.0%) | 200 (83.7%) | 1.00 | 0.023 | 664.3 | 681.0 | 0.097 |
|  |  | G/G | 60 (25.0%) | 39 (16.3%) | **0.59 (0.38-0.93)** |  |  |  |  |
|  | Over-dominant | A/A-G/G | 128 (53.3%) | 121 (50.6%) | 1.00 | 0.620 | 669.3 | 686.0 |  |
|  |  | A/G | 112 (46.7%) | 118 (49.4%) | 1.10 (0.76-1.57) |  |  |  |  |
|  | Log-additive | --- | --- | --- | **0.75 (0.58-0.97)** | 0.026 | 664.5 | 681.2 |  |
| *rs7779895* | Codominant | C/C | 80 (35.1%) | 69 (30.9%) | 1.00 | 0.650 | 631.9 | 652.5 |  |
|  |  | C/A | 106 (46.5%) | 112 (50.2%) | 1.22 (0.80-1.86) |  |  |  |  |
|  |  | A/A | 42 (18.4%) | 42 (18.8%) | 1.14 (0.66-1.95) |  |  |  |  |
|  | **Dominant** | C/C | 80 (35.1%) | 69 (30.9%) | 1.00 | 0.370 | 630.0 | 646.5 | 0.432 |
|  |  | C/A-A/A | 148 (64.9%) | 154 (69.1%) | 1.20 (0.81-1.78) |  |  |  |  |
|  | Recessive | C/C-C/A | 186 (81.6%) | 181 (81.2%) | 1.00 | 0.970 | 630.8 | 647.3 |  |
|  |  | A/A | 42 (18.4%) | 42 (18.8%) | 1.01 (0.63-1.62) |  |  |  |  |
|  | Over-dominant | C/C-A/A | 122 (53.5%) | 111 (49.8%) | 1.00 | 0.420 | 630.2 | 646.6 |  |
|  |  | C/A | 106 (46.5%) | 112 (50.2%) | 1.17 (0.80-1.69) |  |  |  |  |
|  | Log-additive | --- | --- | --- | 1.09 (0.83-1.41) | 0.540 | 630.4 | 646.9 |  |
| *rs17142875* | Codominant | A/A | 74 (30.7%) | 66 (27.4%) | 1.00 | 0.410 | 674.0 | 694.9 |  |
|  |  | G/A | 105 (43.6%) | 120 (49.8%) | 1.27 (0.83-1.94) |  |  |  |  |
|  |  | G/G | 62 (25.7%) | 55 (22.8%) | 0.99 (0.60-1.62) |  |  |  |  |
|  | Dominant | A/A | 74 (30.7%) | 66 (27.4%) | 1.00 | 0.440 | 673.2 | 689.9 |  |
|  |  | G/A-G/G | 167 (69.3%) | 175 (72.6%) | 1.17 (0.79-1.73) |  |  |  |  |
|  | Recessive | A/A-G/A | 179 (74.3%) | 186 (77.2%) | 1.00 | 0.460 | 673.3 | 690.0 |  |
|  |  | G/G | 62 (25.7%) | 55 (22.8%) | 0.85 (0.56-1.30) |  |  |  |  |
|  | **Over-dominant** | A/A-G/G | 136 (56.4%) | 121 (50.2%) | 1.00 | 0.180 | 672.0 | 688.7 | 0.315 |
|  |  | G/A | 105 (43.6%) | 120 (49.8%) | 1.28 (0.89-1.83) |  |  |  |  |
|  | Log-additive | --- | --- | --- | 1.01 (0.79-1.29) | 0.960 | 673.8 | 690.5 |  |
| *rs4727911* | Codominant | G/G | 65 (27.7%) | 67 (27.7%) | 1.00 | 0.630 | 667.2 | 688.0 |  |
|  |  | G/T | 106 (45.1%) | 118 (48.8%) | 1.07 (0.69-1.64) |  |  |  |  |
|  |  | T/T | 64 (27.2%) | 57 (23.6%) | 0.86 (0.52-1.41) |  |  |  |  |
|  | Dominant | G/G | 65 (27.7%) | 67 (27.7%) | 1.00 | 0.960 | 666.1 | 682.8 |  |
|  |  | G/T-T/T | 170 (72.3%) | 175 (72.3%) | 0.99 (0.66-1.48) |  |  |  |  |
|  | **Recessive** | G/G-G/T | 171 (72.8%) | 185 (76.5%) | 1.00 | 0.360 | 665.2 | 681.9 | 0.445 |
|  |  | T/T | 64 (27.2%) | 57 (23.6%) | 0.82 (0.54-1.25) |  |  |  |  |
|  | Over-dominant | G/G-T/T | 129 (54.9%) | 124 (51.2%) | 1.00 | 0.450 | 665.5 | 682.2 |  |
|  |  | G/T | 106 (45.1%) | 118 (48.8%) | 1.15 (0.80-1.65) |  |  |  |  |
|  | Log-additive | --- | --- | --- | 0.93 (0.72-1.19) | 0.560 | 665.8 | 682.4 |  |
| *rs7795646* | Codominant | A/A | 77 (32.2%) | 67 (28.0%) | 1.00 | 0.550 | 669.2 | 690.0 |  |
|  |  | G/A | 108 (45.2%) | 118 (49.4%) | 1.27 (0.83-1.93) |  |  |  |  |
|  |  | G/G | 54 (22.6%) | 54 (22.6%) | 1.16 (0.70-1.91) |  |  |  |  |
|  | **Dominant** | A/A | 77 (32.2%) | 67 (28.0%) | 1.00 | 0.300 | 667.3 | 684.0 | 0.394 |
|  |  | G/A-G/G | 162 (67.8%) | 172 (72.0%) | 1.23 (0.83-1.82) |  |  |  |  |
|  | Recessive | A/A-G/A | 185 (77.4%) | 185 (77.4%) | 1.00 | 0.990 | 668.4 | 685.1 |  |
|  |  | G/G | 54 (22.6%) | 54 (22.6%) | 1.00 (0.65-1.54) |  |  |  |  |
|  | Over-dominant | A/A-G/G | 131 (54.8%) | 121 (50.6%) | 1.00 | 0.350 | 667.5 | 684.2 |  |
|  |  | G/A | 108 (45.2%) | 118 (49.4%) | 1.19 (0.83-1.70) |  |  |  |  |
|  | Log-additive | --- | --- | --- | 1.09 (0.85-1.40) | 0.510 | 668.0 | 684.6 |  |
| *rs2896298* | Codominant | T/T | 93 (38.4%) | 77 (32.4%) | 1.00 | 0.180 | 669.9 | 690.8 |  |
|  |  | C/T | 100 (41.3%) | 119 (50.0%) | 1.42 (0.95-2.12) |  |  |  |  |
|  |  | C/C | 49 (20.2%) | 42 (17.6%) | 1.03 (0.62-1.72) |  |  |  |  |
|  | Dominant | T/T | 93 (38.4%) | 77 (32.4%) | 1.00 | 0.180 | 669.5 | 686.2 |  |
|  |  | C/T-C/C | 149 (61.6%) | 161 (67.7%) | 1.29 (0.89-1.88) |  |  |  |  |
|  | Recessive | T/T-C/T | 193 (79.8%) | 196 (82.3%) | 1.00 | 0.480 | 670.8 | 687.5 |  |
|  |  | C/C | 49 (20.2%) | 42 (17.6%) | 0.85 (0.54-1.34) |  |  |  |  |
|  | **Over-dominant** | T/T-C/C | 142 (58.7%) | 119 (50.0%) | 1.00 | 0.067 | 667.9 | 684.6 | 0.201 |
|  |  | C/T | 100 (41.3%) | 119 (50.0%) | 1.40 (0.98-2.01) |  |  |  |  |
|  | Log-additive | --- | --- | --- | 1.07 (0.83-1.37) | 0.610 | 671.0 | 687.7 |  |
| *rs1072198* | Codominant | T/T | 206 (86.2%) | 201 (83.4%) | 1.00 | 0.089 | 667.9 | 688.7 |  |
|  |  | T/C | 33 (13.8%) | 37 (15.3%) | 1.20 (0.72-2.00) |  |  |  |  |
|  |  | C/C | 0 (0.0%) | 3 (1.2%) | NA (0.00-NA) |  |  |  |  |
|  | Dominant | T/T | 206 (86.2%) | 201 (83.4%) | 1.00 | 0.310 | 669.7 | 686.4 |  |
|  |  | T/C-C/C | 33 (13.8%) | 40 (16.6%) | 1.30 (0.78-2.15) |  |  |  |  |
|  | **Recessive** | T/T-T/C | 239 (100.0%) | 238 (98.8%) | 1.00 | 0.037 | 666.3 | 683.0 | 0.130 |
|  |  | C/C | 0 (0.0%) | 3 (1.2%) | NA (0.00-NA) |  |  |  |  |
|  | Over-dominant | T/T-C/C | 206 (86.2%) | 204 (84.7%) | 1.00 | 0.530 | 670.3 | 687.0 |  |
|  |  | T/C | 33 (13.8%) | 37 (15.3%) | 1.18 (0.71-1.96) |  |  |  |  |
|  | Log-additive | --- | --- | --- | 1.38 (0.86-2.22) | 0.190 | 668.9 | 685.6 |  |
| *rs17142891* | Codominant | G/G | 89 (37.2%) | 75 (31.8%) | 1.00 | 0.230 | 662.7 | 683.5 |  |
|  |  | G/A | 101 (42.3%) | 119 (50.4%) | 1.38 (0.92-2.08) |  |  |  |  |
|  |  | A/A | 49 (20.5%) | 42 (17.8%) | 1.02 (0.61-1.71) |  |  |  |  |
|  | Dominant | G/G | 89 (37.2%) | 75 (31.8%) | 1.00 | 0.230 | 662.2 | 678.8 |  |
|  |  | G/A-A/A | 150 (62.8%) | 161 (68.2%) | 1.26 (0.86-1.85) |  |  |  |  |
|  | Recessive | G/G-G/A | 190 (79.5%) | 194 (82.2%) | 1.00 | 0.480 | 663.1 | 679.8 |  |
|  |  | A/A | 49 (20.5%) | 42 (17.8%) | 0.85 (0.53-1.34) |  |  |  |  |
|  | **Over-dominant** | G/G-A/A | 138 (57.7%) | 117 (49.6%) | 1.00 | 0.087 | 660.7 | 677.4 | 0.228 |
|  |  | G/A | 101 (42.3%) | 119 (50.4%) | 1.37 (0.95-1.97) |  |  |  |  |
|  | Log-additive | --- | --- | --- | 1.05 (0.82-1.36) | 0.680 | 663.5 | 680.1 |  |
| *rs11983106* | --- | G/G | 220 (92.0%) | 222 (91.7%) | 1.00 | 0.960 | 672.4 | 689.1 |  |
|  |  | G/T | 19 (8.0%) | 20 (8.3%) | 1.02 (0.53-1.97) |  |  |  |  |
| *rs727228* | Codominant | T/T | 86 (35.8%) | 74 (30.6%) | 1.00 | 0.390 | 673.7 | 694.6 |  |
|  |  | A/T | 109 (45.4%) | 124 (51.2%) | 1.32 (0.88-1.98) |  |  |  |  |
|  |  | A/A | 45 (18.8%) | 44 (18.2%) | 1.15 (0.69-1.94) |  |  |  |  |
|  | **Dominant** | T/T | 86 (35.8%) | 74 (30.6%) | 1.00 | 0.210 | 672.0 | 688.7 | 0.315 |
|  |  | A/T-A/A | 154 (64.2%) | 168 (69.4%) | 1.27 (0.87-1.87) |  |  |  |  |
|  | Recessive | T/T-A/T | 195 (81.2%) | 198 (81.8%) | 1.00 | 0.920 | 673.6 | 690.3 |  |
|  |  | A/A | 45 (18.8%) | 44 (18.2%) | 0.98 (0.62-1.55) |  |  |  |  |
|  | **Over-dominant** | T/T-A/A | 131 (54.6%) | 118 (48.8%) | 1.00 | 0.210 | 672.0 | 688.7 | 0.315 |
|  |  | A/T | 109 (45.4%) | 124 (51.2%) | 1.26 (0.88-1.80) |  |  |  |  |
|  | Log-additive | --- | --- | --- | 1.11 (0.86-1.43) | 0.430 | 673.0 | 689.7 |  |

OR, Odds ratio; CI, confidence interval; AIC, Akaike’ information criterion; BIC, Bayesian information criterion; *p_FDR_*: FDR corrected *p* value; **p*< 0.05; NA is not applicable.
